# Exploring the genetic architecture of multiple long-term conditions using a genome-wide association study in the UK Biobank population

**DOI:** 10.1038/s41598-025-27839-4

**Published:** 2025-12-06

**Authors:** Anand Thakarakkattil Narayanan Nair, Miles Witham, Avan A. Sayer, Heather J. Cordell, Ewan R. Pearson, ADMISSION Research Collaborative, Victoria Bartle, Rachel Cooper, Ray Holding, Tom Marshall, Fiona E. Matthews, Paolo Missier, Chris Plummer, Sian M. Robinson, Elizabeth Sapey, Thomas Scharf, Mervyn Singer, James M. S. Wason

**Affiliations:** 1 Population Health and Genomics, School of Medicine, University of Dundee, Dundee, DD1 9SY UK; 2 AGE Research Group, Translational and Clinical Research Institute, Faculty of Medical Sciences, Newcastle University, Newcastle Upon Tyne, UK; 3 NIHR Newcastle Biomedical Research Centre, Newcastle Upon Tyne Hospitals NHS Foundation Trust, Cumbria Northumberland and Tyne and Wear NHS Foundation Trust and Newcastle University, Newcastle Upon Tyne, UK; 4 Population Health Sciences Institute, Faculty of Medical Sciences, Newcastle University, Newcastle Upon Tyne, UK; 5 ADMISSION Research Collaborative, Newcastle Upon Tyne, UK; 6 Institute of Applied Health Research, University of Birmingham, Birmingham, UK; 7 Research and Enterprise Office, University of Hull, Hull, UK; 8 Digital Services, Newcastle Upon Tyne Hospitals NHS Foundation Trust, Newcastle Upon Tyne, UK; 9 PIONEER Hub, University of Birmingham, Birmingham, UK; 10 Institute of Inflammation and Ageing, University of Birmingham, Birmingham, UK; 11 University College London Hospitals NHS Foundation Trust, London, UK; 12 Bloomsbury Institute for Intensive Care Medicine, University College London, London, UK; 13 Biostatistics Research Group, Population Health Sciences Institute, Newcastle University, Newcastle Upon Tyne, UK

**Keywords:** Epidemiology, Risk factors, Genetics research

## Abstract

The prevalence of multiple long-term conditions (MLTC) is increasing. It is essential to develop strategies to prevent and manage MLTC; however, the biological mechanisms underlying MLTC are not yet clearly understood. We used UK Biobank data as part of the ADMISSION research collaborative to identify genetic drivers for MLTC. We used the UK Biobank (UKBB) self-reported illness data to characterise MLTC (defined as two or more long-term conditions) using 51 common disease labels. A genome-wide association study (GWAS) was conducted for MLTC and complex MLTC (complex MLTC was defined as having three or more diseases from the 51 self-reported diseases, with these three diseases additionally belonging to different body systems), and post-GWAS analyses were conducted to explore the genomic loci associated with MLTC. We then undertook a factor analysis on the individual-level disease data to identify the factors contributing to MLTC. We investigated the genomics of these factors using single disease polygenic risk score (PRS) and GWAS. The prevalence of simple MLTC was 33.0% (n = 111,184) and complex MLTC was 11.2% (n = 37,650). The majority (81.3%) of significant SNPs from MLTC GWAS were located in chromosome 6 with most of them in the HLA region. The ‘T cell activation’ pathway and apoptosis signalling pathways were identified in gene-based pathway analysis. Five latent factors were identified through factor analysis with the following underlying characteristics: Factor 1, metabolic disease; Factor 2, mental ill health; Factor 3, cancer; Factor 4, musculoskeletal and inflammation-related traits; Factor 5, digestive system-related diseases. The GWAS and PRS-based analysis validated the characteristics of these factors. The MLTC GWAS, complex MLTC GWAS and factor-based GWAS analyses highlighted the association between HLA genes and MLTC. Further research is needed to disentangle the association between MLTC and the HLA genes, along with the integration of multi-omics data.

**Supplementary Information:**

The online version contains supplementary material available at 10.1038/s41598-025-27839-4.

## Introduction

Multiple long-term conditions (MLTC), also referred to as multimorbidity, is defined as the simultaneous occurrence of two or more diseases in the same individual^1^. A long-term condition refers to a disease with a medical diagnosis, present for 12 months or more, is ongoing or permanent in effect, which requires continued monitoring or treatment, results in increased risk of mortality, reduced quality of life, worsens physical or mental health, rise in treatment burden^2^. Studies previously conducted in UK Biobank show that multimorbidity is significantly associated with higher mortality and lower quality of life^3^. According to a recently published systematic review and meta-analysis, the global prevalence of multimorbidity is 37.5% and shows an upward trend; multimorbidity affects nearly half of individuals aged over 60 years globally^4^. A study carried out in 2018 projected that approximately 17% of the UK adult population will have four or more chronic diseases by 2035^5^. These estimates reveal the current and future burden of MLTC and highlight the need to understand the mechanisms underlying disease accumulation leading to the incidence of MLTC. Most previous MLTC research has explored the association of MLTC with behavioural, lifestyle factors and socio-economic deprivation but very few studies have investigated the mechanisms associated with MLTC incidence. A recent review outlined the potential mechanisms of MLTC in four ways: 1) one disease causes another; 2) treatment of one disease leads to the incidence of the second disease; 3) both diseases share a common biological pathway; 4) both diseases share a common external or environmental exposure^6^.

Genetic studies exploring common biological mechanisms for MLTC initially concentrated on pairs of similar or genetically correlated diseases, like asthma and allergic disease, reporting 38 new genetic loci with 7 of them being novel^7^. Moving beyond pair-wise trait analysis, structural equation modelling approaches have been used to disentangle the complex genetic architecture of correlated diseases. For example, GenomicSEM was applied for the joint analysis of five psychiatric traits, which revealed 27 new single nucleotide polymorphisms (SNP) not detected in univariate analysis. A polygenic risk score (PRS) from the GenomicSEM predicted five psychiatric disorder phenotypes with more accuracy than the PRS from univariate approaches^8^. Whilst this method has merits, it limits the number of diseases for MLTC assessment; studies report that the number of diseases at the individual level varies from 0 to 14 with a median number of 3–5^9,10^.

Considering the mechanisms underlying MLTC disease associations, a recent study examined multiple disease pairs and investigated the common genetic loci associated with both diseases for all pairwise combinations, considering level 2 ICD10 codes as diseases^11^. In this study, Dong et al. showed that 46% of the disease pairs from 439 diseases shared genetic components either at a locus, network or overall genetic architecture level^11^. The pairwise approach helped to identify shared genetic mechanisms between two diseases but limits the biological interpretation of multimorbidity when there is a simultaneous occurrence of more than two diseases. An alternative approach applied ‘TreeLFA’, a topic modelling method, to individual-level disease data to identify clusters of multimorbidity along with disease topics which indicate the disease co-occurrence within an individual. This study identified 11 topics using 100 ICD10 codes in UK Biobank. Each topic-based Genome Wide Association Study (GWAS) reported novel genetic associations for single diseases (ICD10 codes) which improved the risk prediction of those single diseases^12^. The topic modelling approach showed promise for disease prediction but provided limited insights into the biological mechanisms of MLTC incidence.

In the current analysis, we consider MLTC as a highly complex heterogeneous disease, and we apply genome-wide association studies (GWAS) methodology to identify genetic markers of overall MLTC. Additionally, we use exploratory factor analysis to identify the latent variables underpinning the disease labels and explore the genetic underpinnings of these latent factors of MLTCs.

## Methods

### Data source

We used UK Biobank (UKBB) data for this analysis. UK Biobank is a prospective cohort study in the UK comprising more than 500,000 individuals with socio-demographic, lifestyle and genomic data^13^. The anonymized data is available for health research through a secure research analysis platform equipped with a variety of statistical analysis packages. At the time of UKB recruitment, a nurse collects information on medical history which includes self-reported prevalent diseases. These data are available in UKB with a description of cancer and non-cancer self-reported illness (category: 100074, Field ID: 20001, Field ID: 20002). In addition to self-reported diseases, we utilized Polygenic Risk Scores (PRS) for selected diseases (n = 26), available in the UK Biobank under category 300^14^.

### Disease list

Initially, we considered 60 diseases defined by the ADMISSION collaborative^2^ informed by a previous recent paper^15^. We then excluded all infectious diseases (Tuberculosis, HIV, Chronic Lyme Disease, recurrent UTI), congenital abnormalities, diseases which are not recorded in the self-reported illness field (hearing impairment, autism, cystic fibrosis) and labelled end-stage kidney disease as chronic kidney disease. Thus, for the current analysis, we used 51 disease conditions to assess MLTC prevalence and its genetics. Each disease is assigned to 11 body systems (e.g. cardiovascular system, digestive system) (Supplementary File 1).

### MLTC definitions

MLTC was defined as having two or more self-reported diseases from the 51 diseases listed at the time of UK Biobank recruitment (example of MLTC: co-occurrence of hypertension and diabetes). Complex MLTC was defined as having three or more diseases from the 51 self-reported diseases with these three diseases additionally belonging to different body systems^16^. (Example of complex MLTC: co-occurrence of hypertension, osteoarthritis and anxiety). We chose to use self-reported disease conditions, as many chronic diseases do not result in hospitalisation and are therefore not captured in the Hospital Episode Statistics coded in UK Biobank using ICD10 codes, and because the primary care data are only available for approximately half of UK Biobank participants.

### Statistical analysis

We estimated MLTC and complex MLTC prevalence in the study population and assessed the association of MLTC and complex MLTC with the socio-demographic and phenotypic characteristics of the study population.

To investigate the genetics of MLTC we conducted two genome-wide association studies (GWAS) with different case–control definitions (i) MLTC GWAS: cases were individuals with two or more diseases and controls with no or one disease (ii) complex MLTC GWAS: cases were individuals with three or more diseases from different body systems and controls may have different combinations of diseases or no disease. We limited the GWAS population to those with White British ethnicity and we applied a cut-off on minor allele frequency (MAF) < 0.01 and Hardy–Weinberg equilibrium p < 1 × 10^−15^. Each GWAS analysis was adjusted for age, sex and genetic principal components PC1-PC20. For quality assessment of genomic data, PLINK2 was employed^17^. We used REGENIE for the GWAS analysis^18^, REGENIE implements GWAS in two steps; the first step uses genotyped data and splits SNPs into blocks to run a ridge regression across each block, and finally to estimate a predictor for each chromosome. In the second step imputed genetic data are used to conduct association analysis at each measured variant along with the predictor from step one. This two-step approach helps to account for population stratification and relatedness among the study participants. Additional data management and statistical analyses were conducted using the R statistical package^19^. To assess the genomic inflation in GWAS analysis, we estimated the genomic inflation factor (λ).To visualise the phenotypic association of independent significant SNPs, we extracted SNP-phenotype association data from the GWAS catalogue (using FUMA) and plotted them as a word cloud. A Venn diagram was created to compare the genes identified in this analysis with those from a previous MLTC analysis.

For gene-based tests and post-GWAS analysis, the Functional Mapping and Annotation of Genome-wide Association Studies (FUMA) web tool was used. The parameters used in FUMA and other post GWAS analysis details are provided in Supplementary Text 1^20^. For gene set enrichment analysis and pathway analysis we used FUMA and PANTHER (https://pantherdb.org/) by using over-representation analysis (ORA). Fisher’s exact test was used to identify enriched pathways, and false discovery rate (FDR) was used for multiple testing correction; GO pathways with adjusted P values < 0.05 are considered significant.

An exploratory factor analysis was applied to the binary disease outcomes with disease presence indicated by ‘1’ and absence by ‘0’. We excluded the sex-specific diseases ‘endometriosis’ and ‘Hyperplasia of the prostate’ from the disease list and used the other 49 diseases’ prevalence data for this analysis. The factor analysis method used was ‘minres’ minimum residual solution with ‘promax’ factor rotation which allows for correlation between factors. Since the data were dichotomous in nature, a tetrachoric correlation structure was applied for factor analysis^21^. Prior to factor analysis, the optimal number of factors was identified using a scree plot with 5 factors considered as optimal.

Previously derived polygenic risk scores (PRS) of specific diseases (n = 26) were used to assess the nature of latent factors identified through factor analysis. Construction and description of these PRS are described elsewhere^14^. A Linear regression model adjusted for age, sex and principal components was used to test the association of factors with PRS and a p-value < 0.001 was considered a significant association. To validate these findings a separate GWAS was conducted for each identified latent factor, using the factor as a continuous outcome in a linear regression adjusted for age, sex and PC1-PC20. All analyses were conducted on anonymised data and followed relevant guidelines and regulations.

## Results

In the UK Biobank, data from 337,054 White British individuals was available for analysis based on the genomic data availability. The mean age at recruitment was 56.8 (7.99) years and 53.7% were female. The median (IQR) number of self-reported diseases was 1 (2) and 34.8% were free of the listed 51 diseases. The most prevalent diseases among the study population were hypertension (27.9%), osteoarthritis (12.1%) and asthma (11.9%).

### MLTC

The MLTC prevalence was 33.0% (n = 111,184) among the study population. MLTC was significantly higher among the socio-economically deprived groups, those who smoke and those who had a sedentary lifestyle. Those with MLTC were older, had higher BMI, greater hyperglycaemia and had higher levels of triglyceride and C-reactive protein (CRP) (Table 1).

**Table 1:** Characteristics of the study population MLTC group and non-MLTC group.

Variable	N	Non-MLTC N = 225,870^1^	MLTC N = 111,184^1^	p-value^2^
Age (years)	337,054	56 (49, 62)	61 (55, 65)	< 0.001
Sex	337,054			0.2
Female		121,468 (54%)	59,514 (54%)	
Male		104,402 (46%)	51,670 (46%)	
HbA1c (mmol/mol)	321,212	34.7 (32.4, 37.1)	36.2 (33.5, 39.4)	< 0.001
BMI (Kg/m2)	335,963	26.2 (23.8, 29.0)	28.0 (25.2, 31.6)	< 0.001
LDL-c (mmol/L)	320,738	3.60 (3.06, 4.18)	3.37 (2.74, 4.02)	< 0.001
HDL-c(mmol/L)	294,162	1.43 (1.20, 1.71)	1.34 (1.12, 1.62)	< 0.001
Triglyceride (mmol/L)	321,080	1.43 (1.02, 2.07)	1.63 (1.15, 2.32)	< 0.001
CRP (mmol/L)	320,647	1.17 (0.59, 2.38)	1.71 (0.84, 3.54)	< 0.001
Creatinine (mmol/L)	321,179	70 (62, 80)	71 (62, 82)	< 0.001
Number of diseases	337,054	0.00 (0.00, 1.00)	2.00 (2.00, 3.00)	< 0.001
Number of body systems involved	337,054	0.00 (0.00, 1.00)	2.00 (2.00, 3.00)	< 0.001

^1^Median (IQR); n (%).

^2^Wilcoxon rank sum test; Pearson’s Chi-squared test.

#### GWAS MLTC

**Fig. 1:**
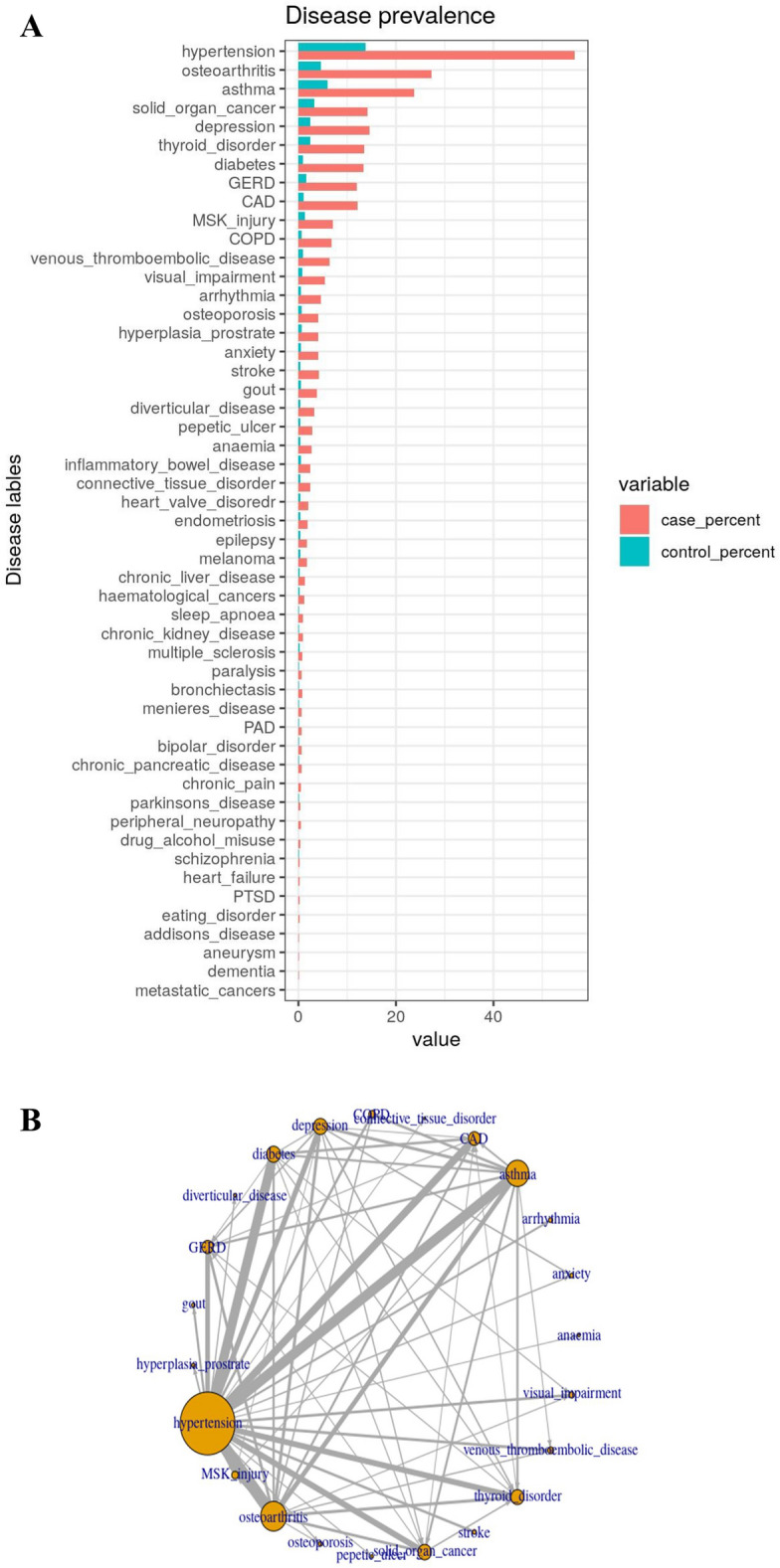
(**A**) Prevalence of disease among MLTC group and non-MLTC group. (**B**) Common pairs of diseases in MLTC population. (Each node indicates disease, and the size of the node indicates prevalence. Edges connecting nodes show the pairs, and the width of edges indicates the prevalence of that pair.)

We undertook a GWAS of MLTC, defined as a case having two or more diseases and a control having one or no diseases (n = 337,054). After quality assessment, 9,243,823 imputed SNPs were included in the analysis. The most prevalent diseases among the cases were hypertension (56.6%), osteoarthritis (27.8%) and asthma (23.7%). Though prevalence estimates were different, hypertension (13.8%), osteoarthritis (5.9%) and asthma (4.5%) were also the most prevalent diseases among controls. The most common disease pair among the MLTC group (> = 2 diseases) was hypertension with osteoarthritis (15.0%) (Fig. 1A&B).

**Fig. 2:**
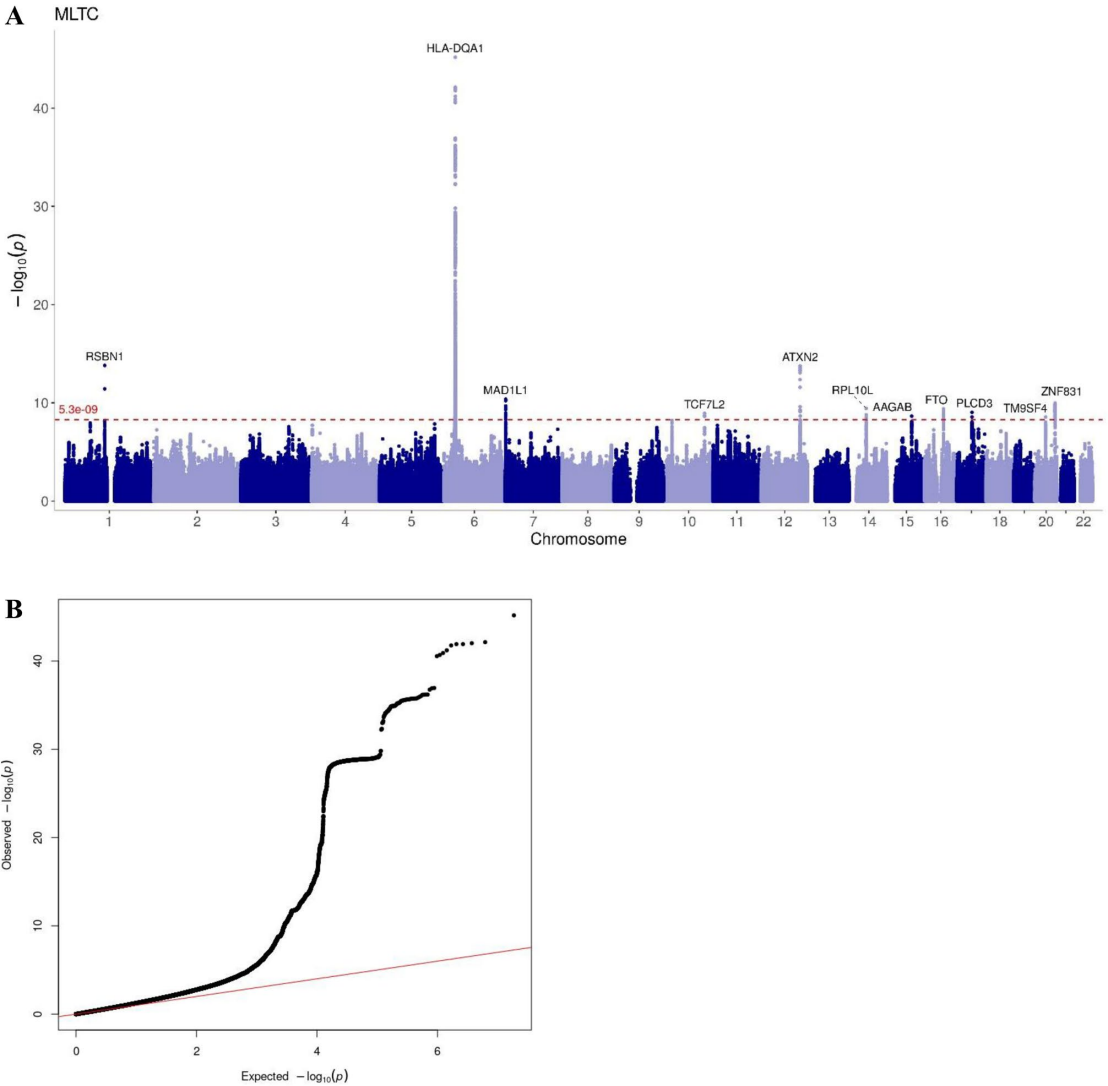
(**a**) Manhattan plot from GWAS of MLTC defined as having 2 or more diseases from the 51 disease list. (**b**) QQ plot from GWAS of MLTC defined as having 2 or more diseases from the 51-disease list.

Figure 2 shows the Manhattan and QQ plots for the MLTC GWAS analysis. There were 166 independent significant SNPs from this GWAS (Supplementary File 2) and most of the significant SNPs (81.3%) were on chromosome 6 with the majority of them positioned in the HLA region. The top significant SNP in chromosome 6 was rs9272539 (OR: 1.08, 95% CI 1.07–1.09, p value = 6.7*10^−46^). More than half of the SNPs were intergenic and 30% were intronic (Supplementary Figs. 2 and 3). From this list, we identified 32 lead SNPs which were independent of each other at r^2^ 0.1. The most significant lead-independent SNP was rs9272539 on chromosome 6; the nearest gene was major histocompatibility complex, class II, DQ alpha 1 (*HLA-DQA1).* The SNP regional plot shows the chromosome 6 region with SNPs with high LD (Supplementary Fig. 4). With respect to our signals on other chromosomes, we identified rs6679677, a SNP located on chromosome 1, which mapped to Round Spermatid Basic Protein 1 (*RSBN1*) / Putative Homeodomain Transcription Factor 1 (*PHTF1*). The SNP located on chromosome 12, rs597808 (GWAS p-value: 1.75*10^–14^), was in the ataxin 2 (*ATXN2*) gene, while rs11766468 on chromosome 7 was mapped to the Mitotic Arrest Deficient 1 Like 1 *(MAD1L1)* gene. A SNP located on chromosome 20 (rs6026728) was mapped to the gene Zinc Finger Protein 831 *(ZNF831)* and rs55872725 was mapped to the *FTO* gene. The SNP on chromosome 10, rs34872471, was mapped to transcription factor 7 like 2 *(TCFL2)* gene.

**Fig. 3:**
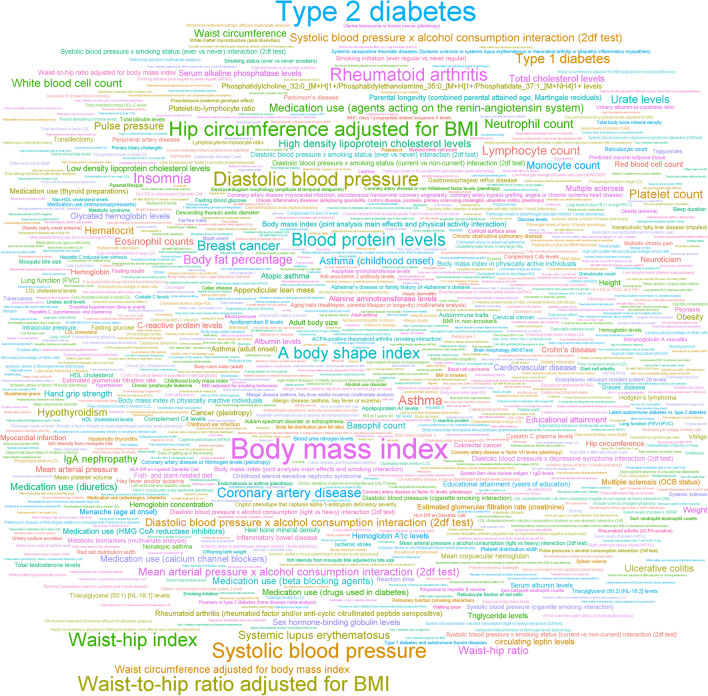
Phenotypic associations of significant SNPs from the GWAS. The word cloud was formed with traits associated with SNPs from this MLTC GWAS, where the size of the word describing the trait is proportional to the number of MLTC GWAS SNPs associated with that trait in published GWAS. [For example, Body Mass Index has been associated with a greater number of SNPs compared to systolic blood pressure].

The word cloud (Fig. 3) showed the phenotypic association of independent significant SNPs . Though BMI, Type 2 diabetes and blood pressure were the most prominent phenotypes in the word cloud, traits related to cancer, inflammation, immune response, mental health, and ageing were also present. Thus, the significant SNPs from MLTC GWAS were related to a variety of physiological processes such as autoimmune response, inflammatory process, metabolic diseases, mental health illness and lifestyle factors, which are likely to underpin MLTC.

#### Gene-based analysis MLTC GWAS

The GWAS summary statistics were functionally annotated using FUMA, which identified 20 genomic risk loci, including 32 lead SNPs and 166 independent significant SNPs (p < 5 × 10⁻⁸). A total of 8,956 candidate SNPs in linkage disequilibrium (LD) with lead SNPs were annotated, of which 7,998 were present in the original GWAS summary data. Using positional and eQTL-based mapping strategies, 199 unique genes (one gene represented by multiple Ensembl IDs) were mapped to these genomic loci.

To visualize the mapped genes, a gene-based Manhattan plot was derived using FUMA (Supplementary Fig. 5) and 199 genes were significantly associated with prevalent MLTC, with ~ 70% of them located in chromosome 6 (Supplementary Fig. 5). Of these 199 genes, 128 (64.3%) were protein-coding and 98 of these protein-coding genes were also located in chromosome 6. In a gene set analysis, using over-representation analysis (ORA) and Protein ANalysis THrough Evolutionary Relationships (PANTHER) gene ontology-based pathway (PANTHER pathways), the ‘*T cell activation’* pathway was overrepresented (FDR p-value: 1.89*10^−2^ and Enrichment Ratio 10.48) and five genes (HLA-DQA1, HLA-DQA2, HLA-DMA, HLA-DMB and HLA-DRA) were overlapping with this pathway’s genes. Similarly, the *‘Apoptosis signalling pathway’* was also overrepresented (FDR p-value: 4.77*10^−2^ and Enrichment Ratio 7.39) with five overlapping genes (*ATF6B, LTA, HSPA1A, HSPA1B and HSPA1L)*. This indicates these genes play a role in the immune process and the processes related to cell death, which is affecting MLTC. Results from a pair-based multimobidity analysis was compared with MLTC GWAS results using a Venn diagram (Supplementary Fig. 6).

Gene set enrichment analysis using GO biological process from MSigDB (https://www.gsea-msigdb.org/gsea/msigdb/index.jsp) mainly identified immune-related biological processes such as “antigen processing and presentation of peptide antigen” (adj. p value 7.56*10^–15^), “antigen processing and presentation” (adj.p value 1.09*10 ^-13^) “peptide antigen assembly with MHC protein complex” (adj. p value 2.06*10^–9^) and “lymphocyte mediated immunity” (adj. p value 1.15*10^–8^). (Supplementary Fig. 7).

### Complex MLTC

The prevalence of complex MLTC was 11.2% (n = 37,650) in the study population. Similar to analyses of simple MLTC, individuals who were older, more obese, and who had hyperglycaemia had higher rates of complex MLTC (Supplementary Table 1). Males had a higher prevalence of complex MLTC, whilst MLTC prevalence was similar across both sexes. About 57.1% of study participants were affected with diseases of the cardiovascular system, 28.6% had diseases related to the musculoskeletal system and 22.5% had the respiratory system-related illness.

#### Complex MLTC GWAS

In the complex MLTC GWAS, cases were defined as having three or more diseases which belong to three different body systems and controls were individuals who might have up to three diseases but not from different body systems. The most prevalent diseases amongst cases were hypertension (65.5%), osteoarthritis (39.4%) and asthma (32.6%) (Supplementary Fig. 8A). Thus, the most affected body systems in cases were the cardiovascular, musculoskeletal and respiratory systems. In controls, hypertension (23.2%), asthma (9.2%) and osteoarthritis (8.6%) also had high prevalence. The most commonly involved disease systems were cardiovascular, musculoskeltal and metabolic and endocrine systems, second common system triad was cardiovascular, musculoskeletal and respiratory systems (Supplementary Fig. 8B).

**Fig. 4:**
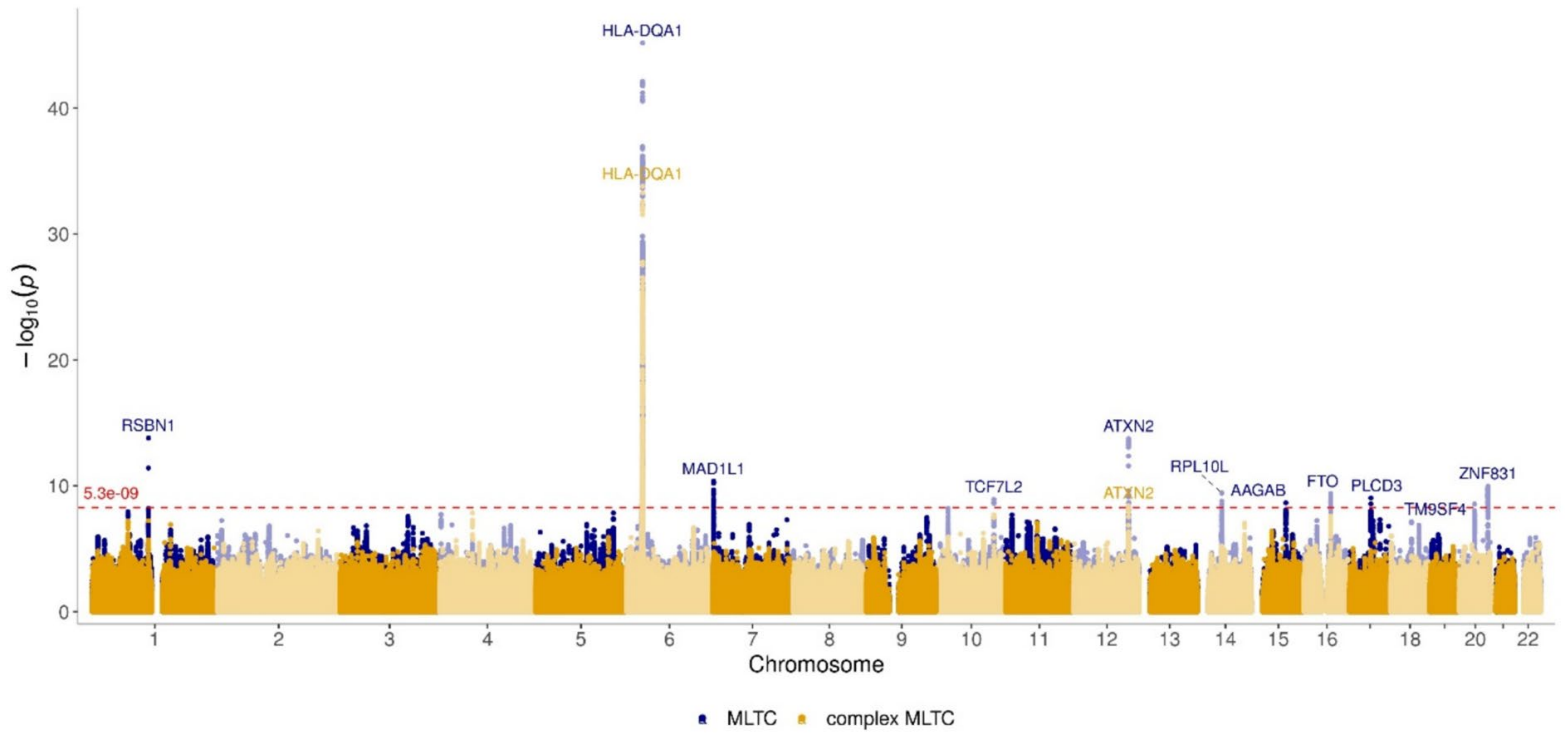
Manhattan plot comparing MLTC GWAS and complex MLTC GWAS (Blue colour indicates the SNPs from the MLTC GWAS and yellow colour indicates the SNPs from Complex MLTC GWAS).

The Manhattan plot from the complex MLTC GWAS is shown in Supplementary Fig. 9 and a plot comparing the results with those from the MLTC GWAS is shown in Fig. 4. From the complex MLTC GWAS, 78 independent significant SNPs were identified and 8 lead independent SNPs were detected from this list (r^2^ 0.1), details are given in Supplementary File 3. More than half of these SNPs were intergenic in nature and located on chromosome 6 (Supplementary Figs. 10 & 11). *HLA-DQA1* was the nearest gene for the lead-independent significant SNP (*rs9272539)* and the lead SNP on chromosome 12 *(*rs35350651) was mapped to *ATNX2.* Similarly, lead SNPs on chromosomes 10 and 16 were mapped to *TCFL2* and *FTO*. These findings were comparable to the MLTC GWAS results.

#### Gene-based analysis complex MLTC GWAS

From the complex MLTC GWAS results, 132 genes were mapped using FUMA and out of these genes 86 (65.2%) were protein-coding genes and on chromosome 6. Compared to MLTC GWAS, 122 genes were common in both GWAS, and 16 genes were present only in complex MLTC GWAS (*TRIM26, HCG17, HCG18, HCG20, MUC22, C6orf15, CDSN, TCF19, RNU6-850P, SAPCD1, SAPCD1-AS1, VARS, DXO, TNXA, HNRNPA1P2 and HLA-DOA*).

To assess the HLA region’s association with MLTC and complex MLTC and to assess what diseases were contributing to this strong signal, we conducted GWAS analysis adjusting for individual diseases. A GWAS of MLTC and complex MLTC adjusted for age, sex, principal components, diabetes, asthma, and thyroid disorder removed the statistical significance of the HLA region (Supplementary Figs. 12 and 13), suggesting that the strong association seen with HLA in the unadjusted analyses may be largely driven by the known HLA associations with these diseases.

The genomic inflation factor (λ) for MLTC GWAS was 1.2, and for complex MLTC GWAS, it was 1.1. The GWAS summary statistics were adjusted for λ, and adjusted p-values were used to perform sensitivity analysis. This analysis also showed *HLA-DQA1* as the top significant gene, most genes were intergenic, and gene set enrichment analysis also reported “antigen processing and presentation of peptide antigen” and “antigen processing and presentation” (Supplementary File 4).

### Exploratory factor analysis

**Fig. 5:**
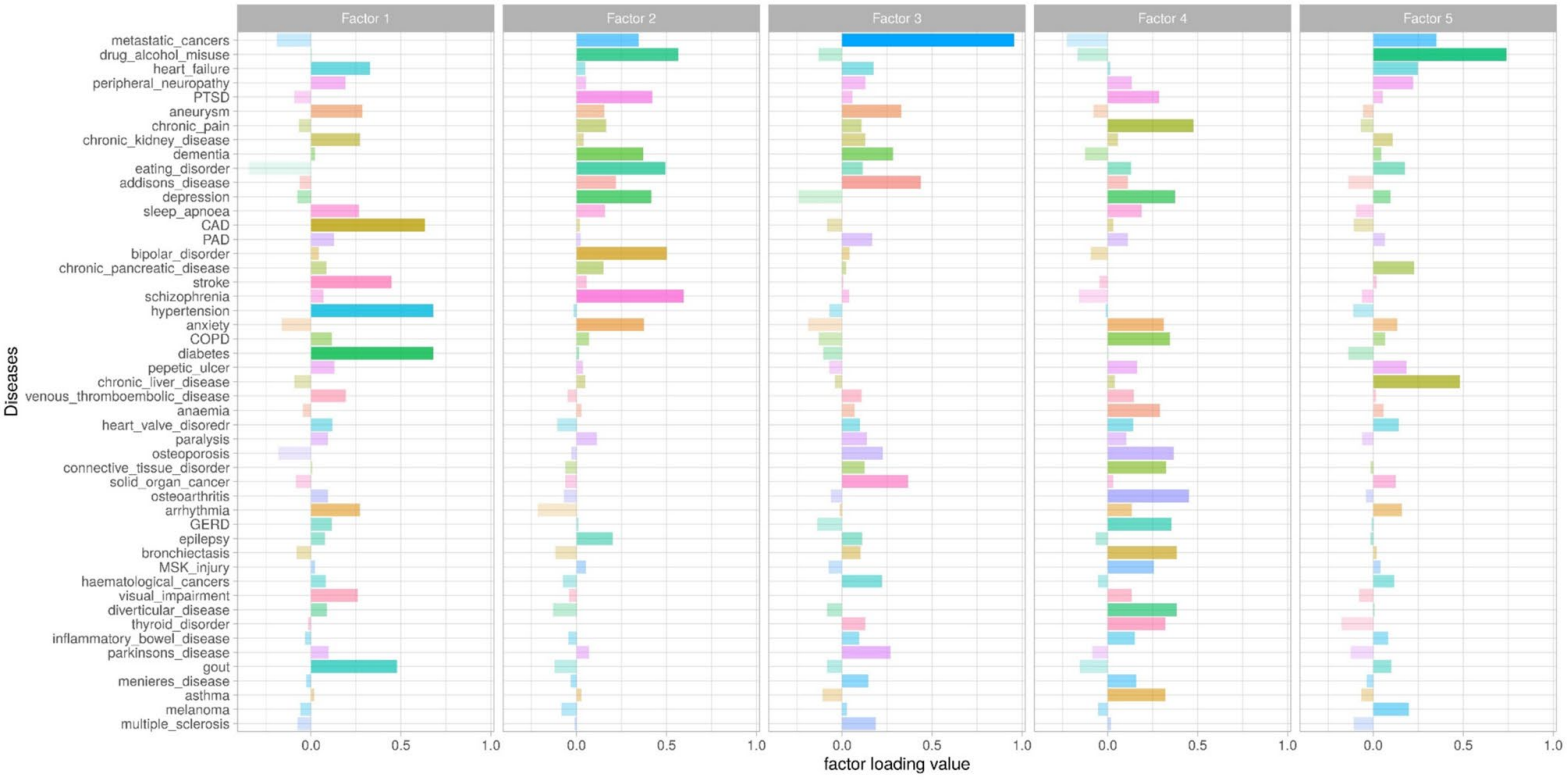
Factor loading and diseases (the X-axis shows the factor loading value and the Y-axis shows diseases). (PTSD: post-traumatic stress disorder, CAD: coronary artery disease, PAD: peripheral arterial disease, COPD: chronic obstructive pulmonary disease, GERD: gastro-oesophageal reflux disease, MSK_injury: musculoskeletal injury).

Prior to the application of exploratory factor analysis, the suitability of the disease data for factor analysis was assessed using Bartlett’s test of sphericity (Chisq = 88,464.40, p < 0.001) and Kaiser Meyer Olkin (KMO) overall measure of sampling adequacy (KMO = 0.61). Both measures indicated the appropriateness of the data for the factor analysis. A five-factor structure was suggested based on scree plot visualization (Supplementary Fig. 14). Factor analysis resulted in five identified factors with factor loadings shown in Fig. 5.

Based on the factor loadings we characterized each factor. Factor 1 was mainly loaded with metabolic diseases (hypertension, diabetes, gout, chronic kidney disease and coronary artery disease). Factor 2 was loaded with mental illness-related diseases: bipolar disorder, drug alcohol misuse, schizophrenia and eating disorders. Cancer, connective tissue disorders, Parkinsonism and Addison’s disease were loaded in Factor 3. Factor 4 had musculoskeletal/ inflammatory related diseases, chronic pain, osteoarthritis, COPD and asthma. Factor 5 was loaded with digestive system-related diseases, mainly chronic liver disease, chronic pancreatic disease, and metastatic cancer. Some diseases or conditions were loaded in multiple factors like ‘alcohol misuse’ in Factor 2 (mental health-related) and Factor 5 (digestive system-related). All factors were positively correlated with the number of self-reported diseases (Supplementary Fig. 15).

To review the nature and characteristics of each factor we regressed each factor on specific disease polygenic risk scores (PRSs) adjusted for age, sex and genetic principal components using linear regression. Factor 1 (metabolic diseases) was strongly and significantly associated with hypertension and T2D PRSs, while Factor 2 (mental ill health) was associated with schizophrenia and bipolar disease PRSs. Factor 3 (autoimmune) was associated with type 1 diabetes, multiple sclerosis, and venous thromboembolic disease PRSs. Factor 4 (inflammatory/musculoskeletal) was strongly associated with the asthma, rheumatoid arthritis, type 2 diabetes PRSs. Factor 5 was mainly loaded with alcohol use and chronic liver disease, chronic pancreatic disease, and arrhythmia. In PRS analysis, atrial fibrillation PRS and colorectal cancer PRSs showed significant associations with factor 5 (Supplementary Fig. 16). These positive relations with specific disease PRS show the characteristics of MLTC Latent factors identified from the factor analysis.

#### GWAS of Latent factors identified from exploratory factor analysis

*GWAS of Factor 1:* Factor 1 was mainly loaded with metabolic diseases like hypertension, diabetes, and CAD phenotypically. Based on PRS analysis hypertension, T2D, stroke and atrial fibrillation, polygenic risk scores were significantly associated with Factor 1. The Manhattan plot Factor 1 GWAS given in Supplementary Fig. 17. The lead SNP from this GWAS, r*s7903146*, positioned in chromosome 10 and nearest gene was *TCF7L2*. The SNP rs111338191 was mapped to the *LINC02356* gene and rs1421085 SNP was mapped to *FTO* gene. Chromosome 9 had a significant SNP rs10811652 mapped to *CDKN2B antisense RNA 1 ( CDKN2B-AS1)*. Most of the SNPs are intergenic in nature and the majority are located on chromosome 6. There were 23 lead-independent significant SNPs from this GWAS, and these SNPs are mapped to 138 genes. All these findings support the metabolic features of factor 1, and both PRS and GWAS analysis confirm this.

*GWAS of Factor 2:* Factor 2 was mainly associated with bipolar disorder and schizophrenia phenotypically and in PRS-based analysis. Although PRS analysis suggested the factors features are related to mental health illness, the GWAS analysis did not identify any significant SNPs. GWAS results are given in Supplementary Fig. 18.

*GWAS of Factor 3:* Factor 3 had a high loading of cancer, multiple sclerosis, and Parkinsonian syndromes. The results of the GWAS analysis are presented in Supplementary Fig. 19*.* The SNP located on chromosome 6, rs3094228 which is mapped to gene HLA complex P5 *(HCP5)*. A SNP on chromosome 2 (rs148374241) was mapped to tousled like kinase 1 (*TLK*1) gene, which is involved in cancer genesis. In PRS-based analysis, factor 3 was associated with diseases which have autoimmune pathophysiology, like type 1 diabetes (T1D), multiple sclerosis, ulcerative colitis (UC), and cancer.

*GWAS of Factor 4:* Factor 4 is related to respiratory and musculoskeletal MLTCs with high loading of asthma, COPD, chronic pain, and osteoarthritis. The lead SNP (rs9272426) from GWAS analysis (Supplementary Fig. 20*)* was present on chromosome 6, in the *HLA DQA1* gene. The second lead SNP rs2476601 was mapped to the protein tyrosine phosphatase non-receptor type 22 *(PTPN22)* gene*.* The PRS-based analysis had a highly significant association with asthma PRS, T2D PRS and rheumatoid arthritis PRS.

GWAS Factor 5: Chronic liver and pancreatic diseases, alcohol misuse, arrhythmia and cancer were loaded in Factor 5. The GWAS analysis failed to identify any significant genomic loci associated with factor 5 (Supplementary Fig. 21*).* The PRS-based analysis showed a significant association with atrial fibrillation, melanoma and colorectal cancer PRS.

Based on the factor GWAS analysis, three latent factors (factor 1, factor 3 and factor 4) had evidence to support the phenotypic disease loading of each factor and in PRS analysis all five factors showed significance to corresponding factors loading. Details of the significant SNPs from the factor GWAS are given in Supplementary File 5.

## Discussion

MLTC pose a great challenge to healthcare systems across the globe, particularly in the context of ageing populations^22^. Although we understand many of the phenotypic and sociodemographic drivers for MLTC incidence, the genetic factors associated with MLTC are less clear. This analysis explored the complex genetic underpinning of MLTC, and it differs from approaches like multi-trait analysis of GWAS (MTAG) in two ways. The first way was conducting a GWAS of overall MLTC by considering MLTC as a highly complex heterogeneous disease condition. The second way was by dissecting the self-reported morbidity data using exploratory factor analysis and conducting GWAS and PRS-based analysis of the latent factors to reveal the genetic associations of different MLTC latent factors.

From the simple MLTC GWAS analysis, we identified 128 protein-coding genes associated with MLTC. The major genes identified from MLTC GWAS (*HLA-DQA1, PHTF1, RSBN1, ATXN2*, *MAD1L1, ZNF831, FTO, TCFL2)* played a significant role in ageing and lifespan^23^, rheumatoid arthritis and other autoimmune diseases^24,25^, multiple neurodegenerative diseases^26^, several MLTC risk factors (smoking, educational attainment) and diseases (schizophrenia and depression)^27–30^, antihypertensive drug use and cardiovascular diseases^31^, obesity mechanisms^32^, diabetes risk^33^. The 16 genes present only in complex MLTC GWAS, compared to MLTC GWAS, were involved in immune and inflammatory response, cancer, and diabetes pathophysiology^34–37^. These associations shows the strength of MLTC GWAS approach.

The gene set enrichment analysis based on MsgDB primarily showed immune and inflammation-related biological processes. Based on PANTHER Overrepresentation Test using the gene set from MLTC GWAS showed a significant overlap with ’T cell activation’ and ‘Apoptosis signalling’ pathways. A defective apoptosis of immune cells could trigger autoimmune diseases, similarly, a defective apoptosis could lead to neurodegenerative, cardiac, hepatic and renal diseases^38^, inflammatory diseases and cancer^39,40^. T-cell activation mediates immune responses and leads to autoimmune diseases and tumour development^41^. This genetic analysis suggests that apoptosis signalling and T cell activation may be a common underlying mechanism increasing the risk for multiple diseases to co-occur in individuals.

Comparing our study with other recent genetic studies of MLTC, we investigated genetic loci common and unique between our study and that of Dong et al.^11^, which assessed the shared genetics of multiple disease pairs using 439 ICD10 codes used for hospital admission. About ~ 8% (n = 122) of the genes from MLTC GWAS were overlapping with the Dong et al. study. Genes located near the HLA locus mainly constituted the common gene list (n = 13). Thus, previous analyses as well as ours highlight the need to examine the HLA region’s involvement in the cooccurrence of multiple diseases. Dong et al. reported that of all the SNPs reported in relation to disease pairs, 73% of them were in the HLA region of the human genome^11^. In our GWAS of MLTC, more than half of the mapped genes belong to the HLA region and a significant number of genes from factor GWAS were also positioned in the HLA region. Previous studies report the role of *HLA DQA1* and *HLA DRB1* role in autoimmune, mental ill health, behavioural and infectious disease occurrence^42^. These two genes are reported to be most pleiotropic in nature with 31 and 19 disease associations^43^. The GWAS of complex MLTC also highlighted the importance of the HLA region in MLTC incidence. The significance of the HLA region decreased when adjusting the MLTC and complex MLTC GWAS for diabetes, asthma, and thyroid disorders, suggesting that the strong HLA association seen in the unadjusted analyses may be driven by the HLA associations with these diseases. However, we consider that HLA still plays a crucial role in MLTC incidence because when we adjust for these three diverse diseases, we essentially adjust for three main pathways (metabolic, autoimmune, and inflammatory processes) of MLTC itself, suggesting that HLA involvement extends beyond a singular disease pathway.

This analysis identified 73 genes that were not present in the multimorbidity pair analysis of Dong et al. Of these, we highlight the *ASXL1* gene*,* which is related to cognitive function, neuroticism and cancer^44,45^. Similarly, the *NEGR1* gene is associated with obesity and educational attainment^27,46^. These genes found only in our analysis were also related to multiple diseases and MLTC risk factors. The finding that there was only an 8% overlap between our analysis and that of Dong et al. may reflect the larger number of disease codes used by Dong et al. compared to 51 defined disease codes used here, and it may also reflect the difference in the approach used, like considering disease pairs and overall MLTC. Additionally, they used ‘obesity’ and ‘disorders of lipoprotein metabolism’ as disease conditions while we did not include these as diseases in our analysis. The pair-based multimorbidity analysis builds up a list of variants representing shared pathways/physiology of two diseases, while in our GWAS of MLTC, we consider MLTC as a complex disease caused by differing pathophysiological processes such as the metabolic syndrome, oxidative stress, abnormal immune responses and inflammation^47^.

Exploratory factor analysis of the multimorbidity data revealed five factors, each positively correlated to the number of self-reported diseases, with factor 1 having the highest correlation. This factor analysis results were comparable with a study in a Spanish population which determined five multimorbidity patterns labelled as cardio-metabolic, psychiatric-substance abuse, mechanical-obesity-thyroidal, psychogeriatric and depressive^48^. Another study showed different factor patterns across different age groups, with an older population having a four-factor structure^49^. Similar to the factor analysis approach where all diseases are considered simultaneously, latent Dirichlet allocation (LDA)^50^ and latent factor allocation with a tree-structured prior (TreeLFA) was previously applied to identify ‘topics’. Our study was different from these previous studies at the study population level (with a slightly older population) and at the analysis level where we have explored the genetics of the latent factors.

The factor GWAS highlighted characteristics of each factor: factor 1 showed associations with SNPs located near genes (*TCF7L2*, *LINC02356*, *FTO, CDKN2B-AS1);* these genes are reportedly involved in pathogenesis of diabetes^51^, smoking and blood pressure^51^, obesity^32^, cardiovascular disease^52^. Factor 3 was related to genes (*HCP5, TLK*1) which played a role in autoimmune diseases^53^, cancer incidence^54^ and cancer genesis via replication stress and DNA damage in cancer cells^55,56^. Genes involved in autoimmune disease origins and human longevity^23,57^, rheumatoid arthritis development^58^ were related to Factor 4 (*HLA DQA1, PTPN22).*The common signal in the GWAS of the latent factors was also HLA (GWAS factor 1, factor 3 and factor 4) suggestive of the involvement of HLA in MLTC incidence.

The strength of this analysis includes the use of different MLTC definitions and the use of multiple methods to explore MLTC genetics (GWAS and factor analysis). The main limitations of this analysis were that we used self-reported disease rather than clinical diagnostic codes, which might introduce bias regarding missed disease diagnosis, resulting in misclassification. We chose to use self-reported disease, as using hospital episode statistics is biased to diseases that result in hospitalisation and the primary care data is only available on approximately half of the UK Biobank population. However, it is reported that the MLTC defined in UKBB primary care data, and UKB baseline assessment data have similar characteristics^59^. Secondly, this study was conducted in UK Biobank which is a relatively healthy, white ethnic population and is not totally representative of the general population.

In summary, we conducted a GWAS analysis of MLTC and complex MLTC and investigated the genetics of latent factors related to MLTC in the UKB data. Though the MLTC definition makes MLTC a heterogenous complex disease, this analysis showed the importance of the HLA region’s complex pleiotropic association with MLTC, which was shown repeatedly in both the MLTC GWAS and the complex MLTC GWAS. Further research using omics is required to unravel the mechanistic pathways associated with the occurrence of MLTC.

## Supplementary Information

Supplementary Information 1.

Supplementary Information 2.

Supplementary Information 3.

Supplementary Information 4.

Supplementary Information 5.

Supplementary Information 6.

## Data Availability

Access to the original data utilized in this analysis is available from the UKBiobank upon appropriate request and approval (https://www.ukbiobank.ac.uk/enable-your-research/apply-for-access).
