# Supplementary material for: Exploring the genetic architecture of multiple long-term conditions using a genome-wide association study in the UK Biobank population
Source: Sci Rep. 2025 Dec 6;15:44096. doi: 10.1038/s41598-025-27839-4 (PMC12715236; doi:10.1038/s41598-025-27839-4)
Supplement: Supplementary file 1 — Supplementary Information 1. [file 41598_2025_27839_MOESM1_ESM.docx]

Supplementary Figure 1: Flow chart selection of individuals for GWAS

Supplementary Figure 2: Functional consequences of SNPs on genes (FUMA) MLTC GWAS


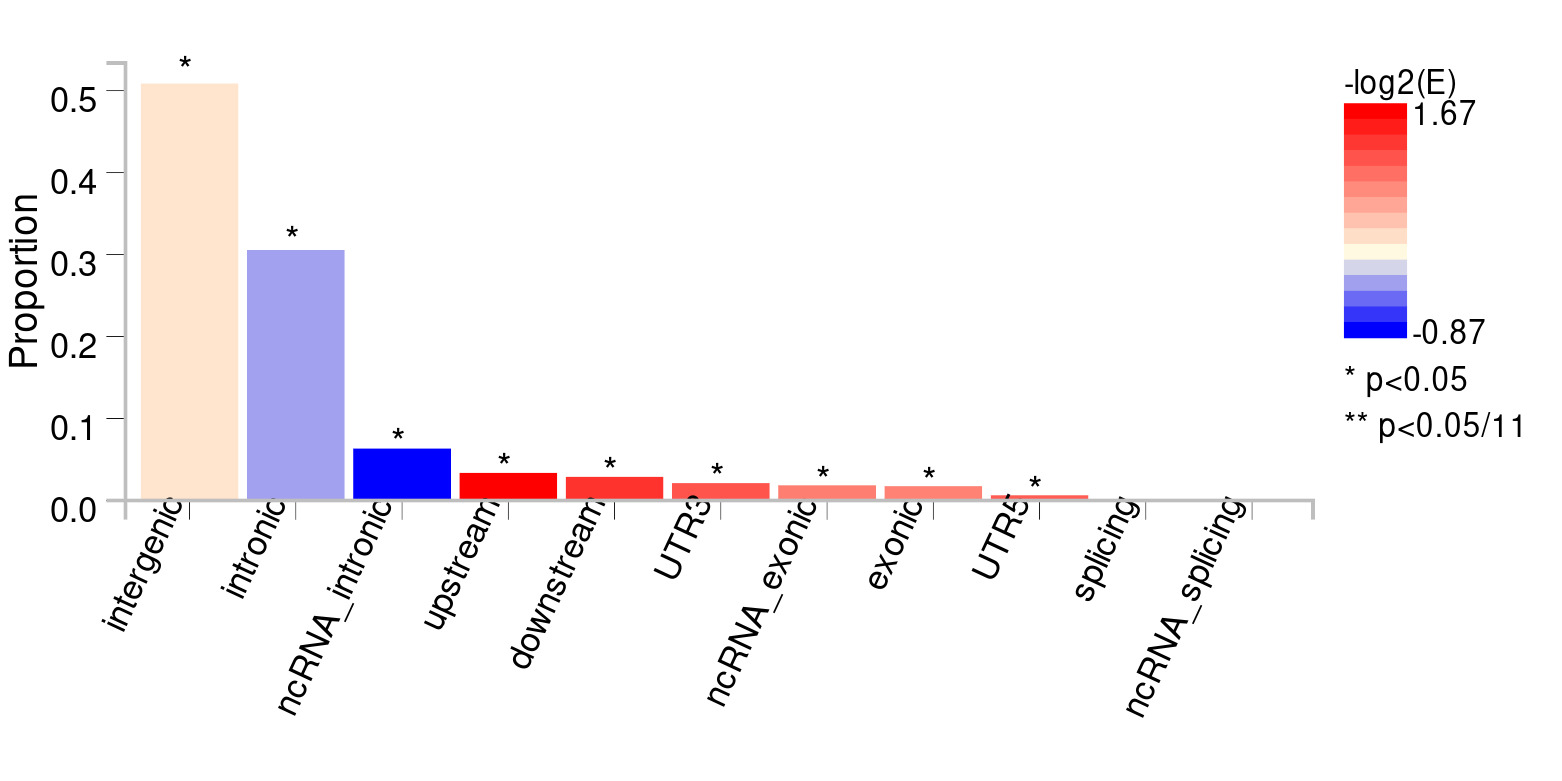


Supplementary Figure 3: Distribution of MLTC GWAS significant SNPs


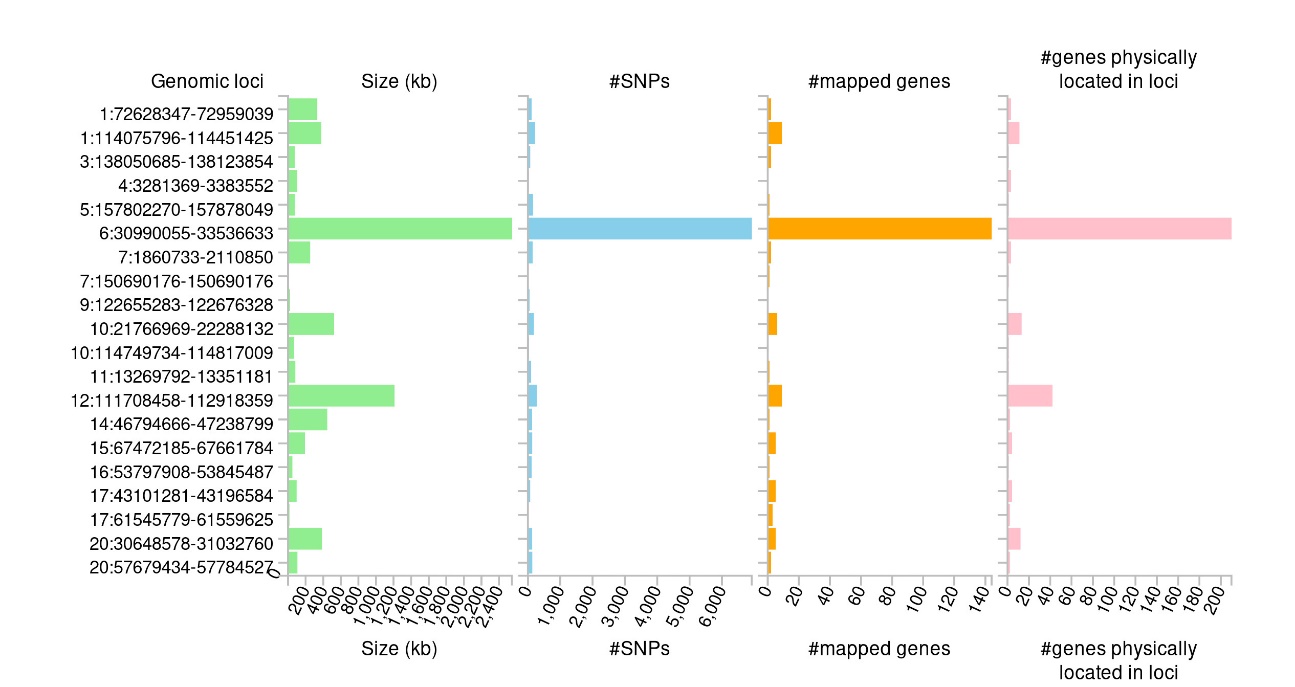


Supplementary Figure 4: Regional plot visualising the lead significant SNP in Chr6


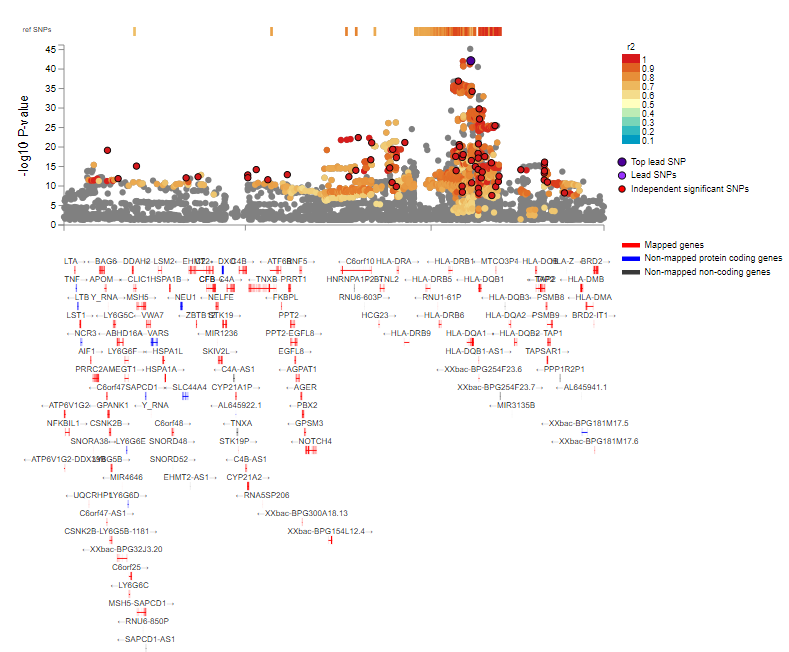


Supplementary Figure 5: Gene-based test Manhattan plot from GWAS of MLTC defined as having 2 or more diseases from 51 disease list. (Input SNPs were mapped to 19077 protein-coding genes. Genome-wide significance (red dashed line in the plot) was defined at P = 0.05/19077 = 2.621e-6). A detailed list of genes and characteristics is given in Supplementary File 2.


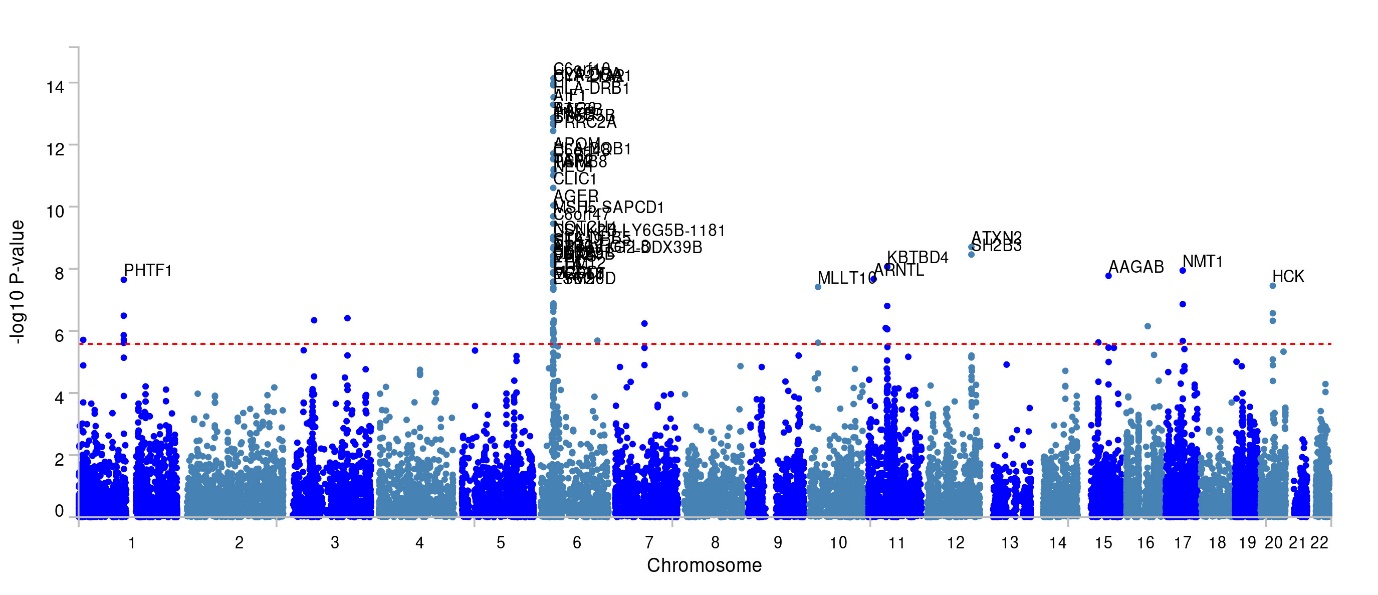


Supplementary Figure 6: Venn diagram comparing genes identified from MLTC GWAS with Dong et al.


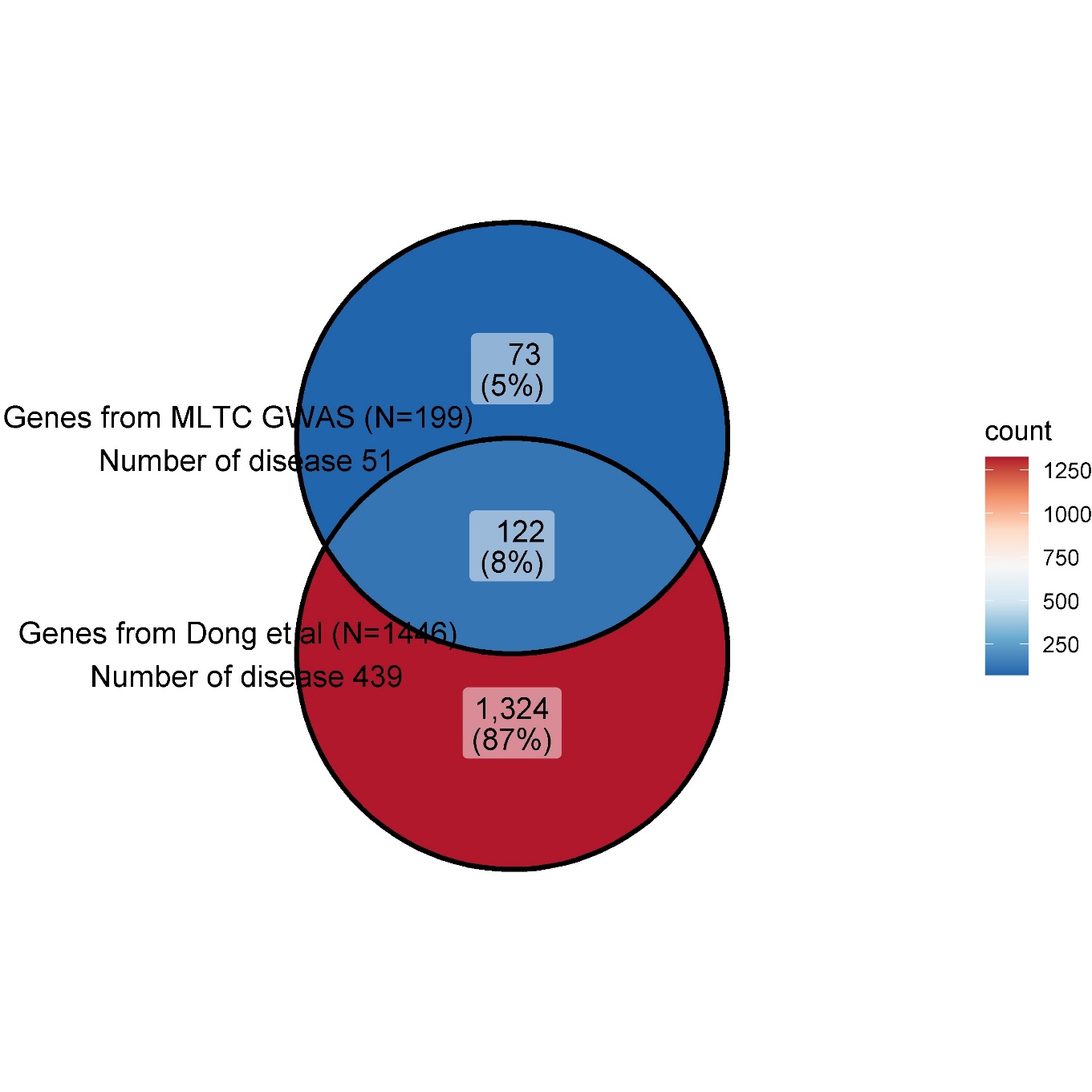


Supplementary Figure 7: Gene set enrichment analysis from FUMA based on MLTC GWAS


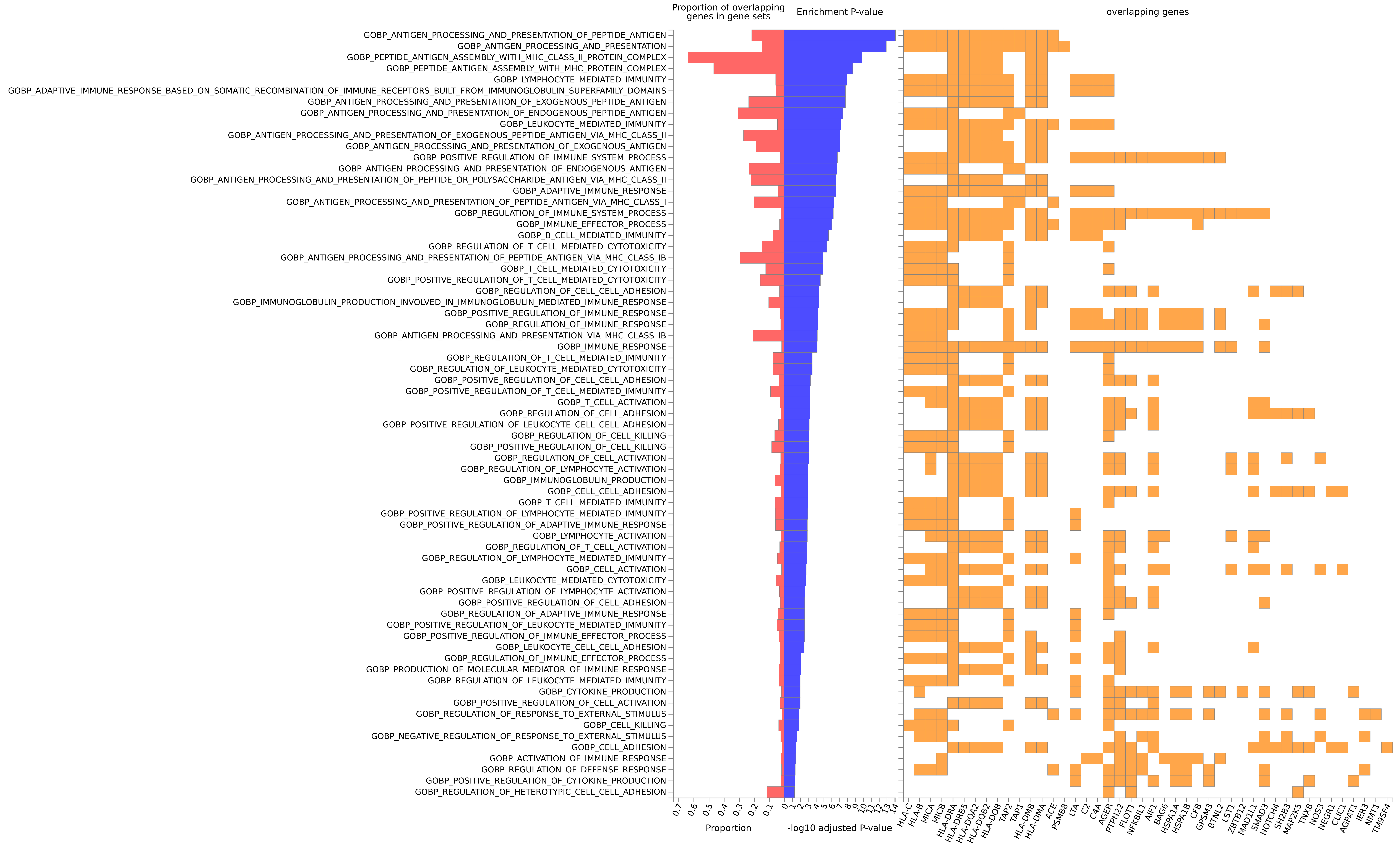


Supplementary Figure 8A: Prevalence of diseases among cases and controls in complex MLTC GWAS.


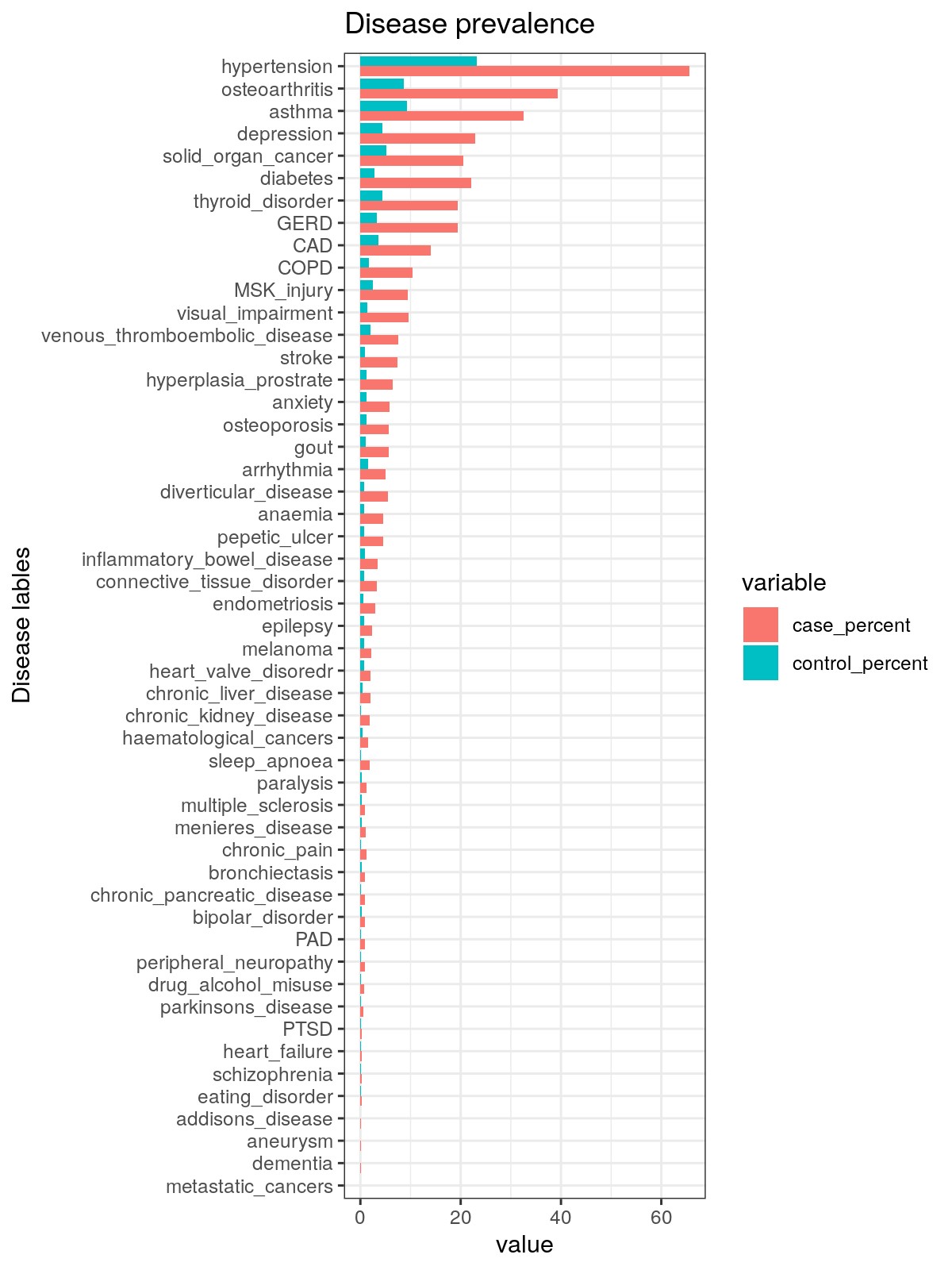


Supplementary Figure 8B: Most common disease system triad in complex MLTC population. (Each dots in the lower deck of the figure indicate each disease system. The intersection size bar chart shows the number of triads observed in the complex MLTC population, and the set size indicates the prevalence of individual disease system in the complex MLTC population).


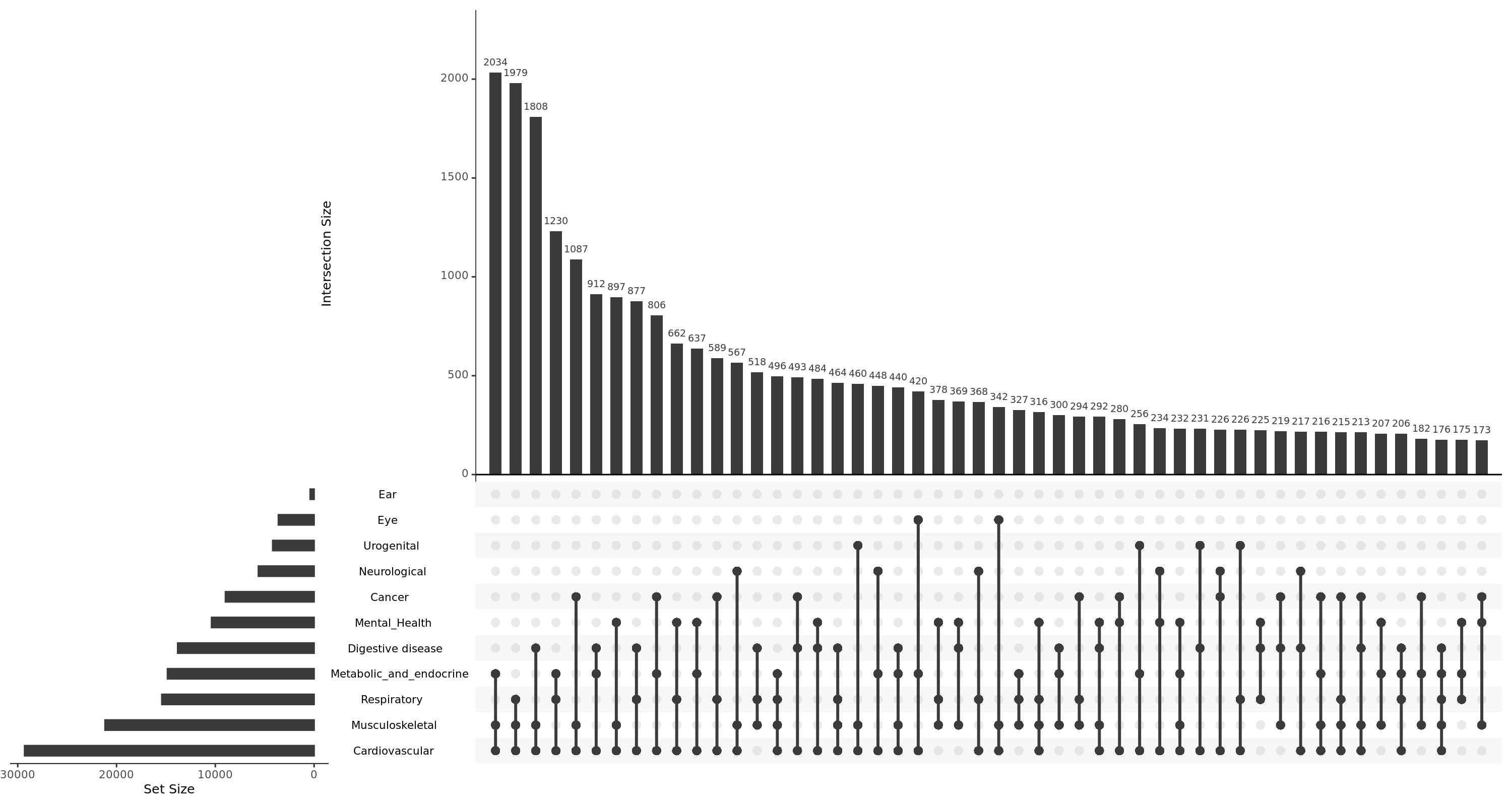


Supplementary Figure 9: Manhattan plot from GWAS of complex MLTC defined as having 3 or more diseases from 51 disease list and these diseases belong to three different body systems.


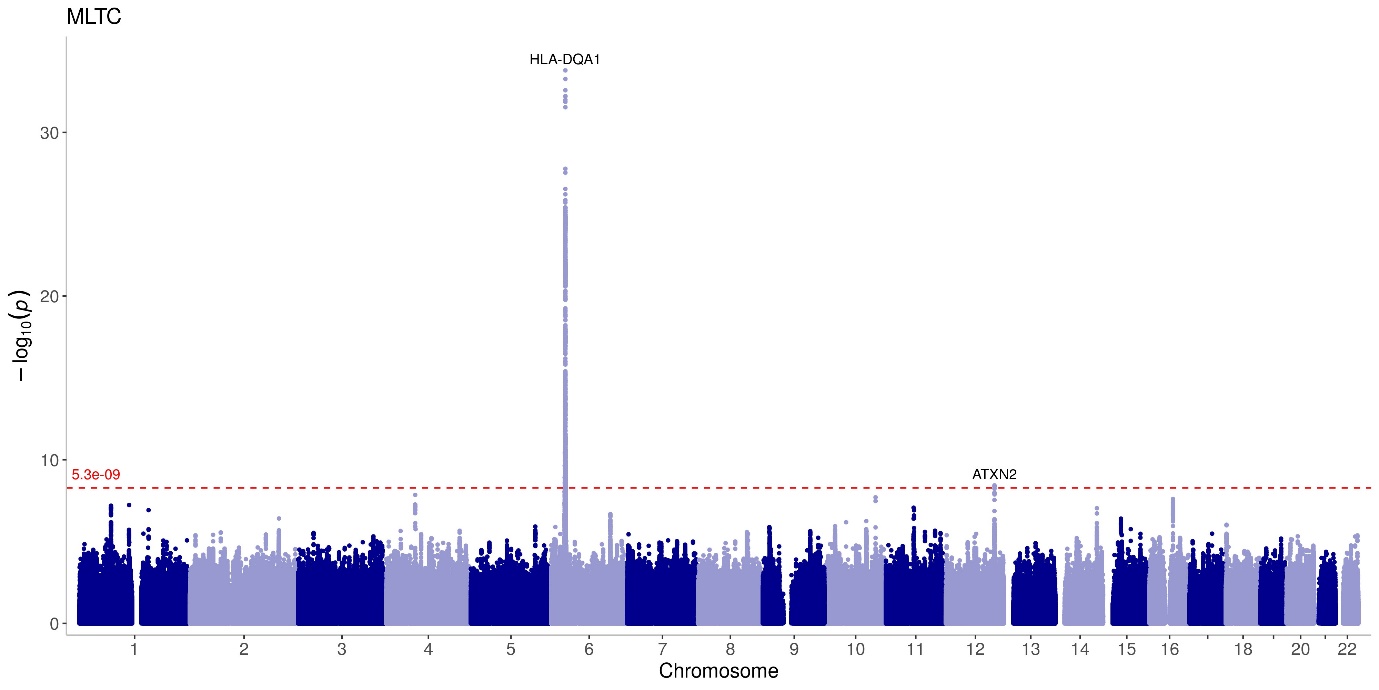


Supplementary Figure 10: Functional consequences of SNPs on genes (FUMA) complex MLTC GWAS


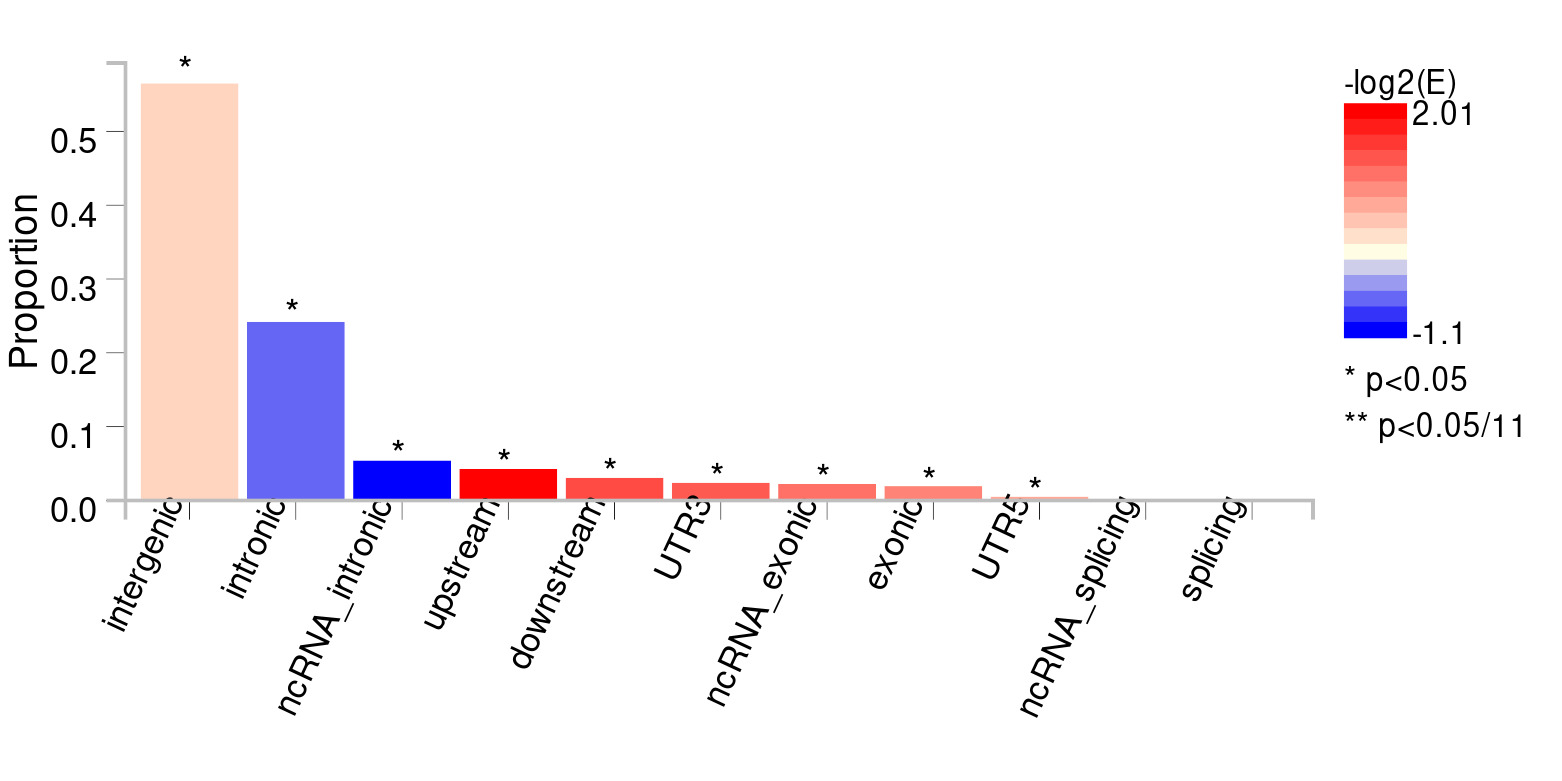


Supplementary Figure 11: Distribution of complex MLTC GWAS significant SNPs


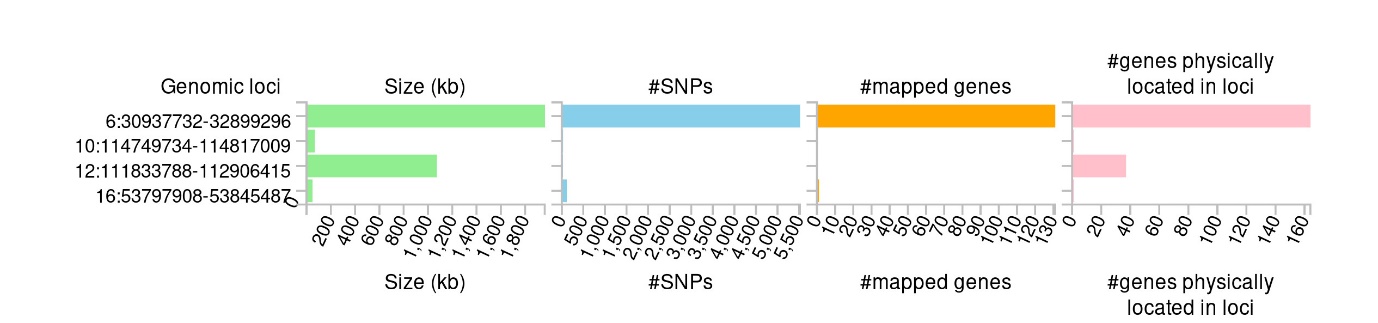


Supplementary Figure 12: Manhattan plot from GWAS of MLTC adjusted for diabetes, asthma and thyroid disorder.


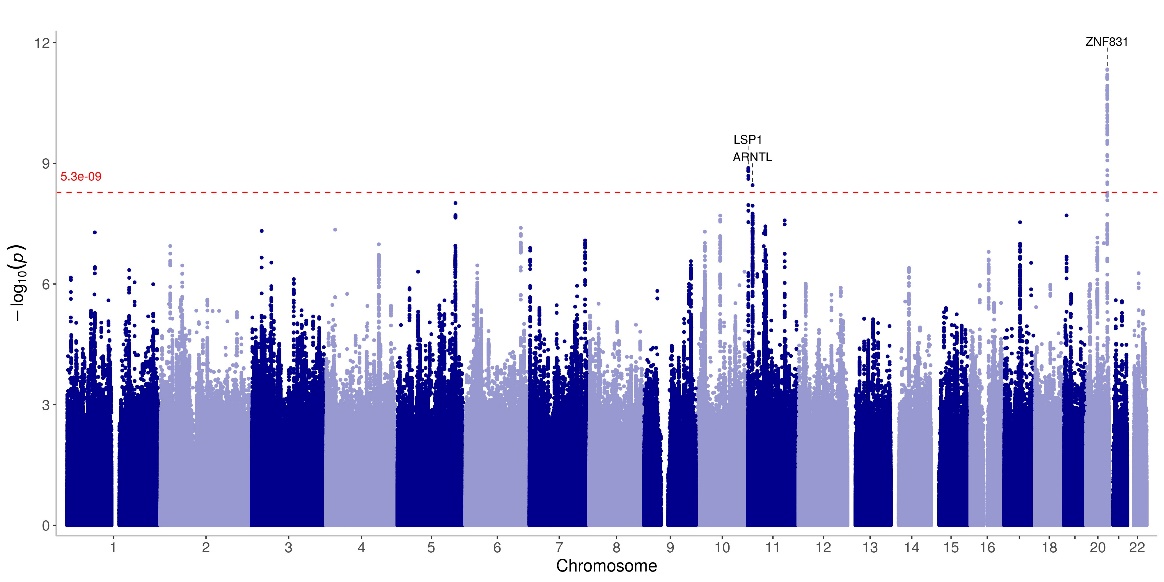


Supplementary Figure 13: Manhattan plot from GWAS of complex MLTC adjusted for diabetes, asthma and thyroid disorder.


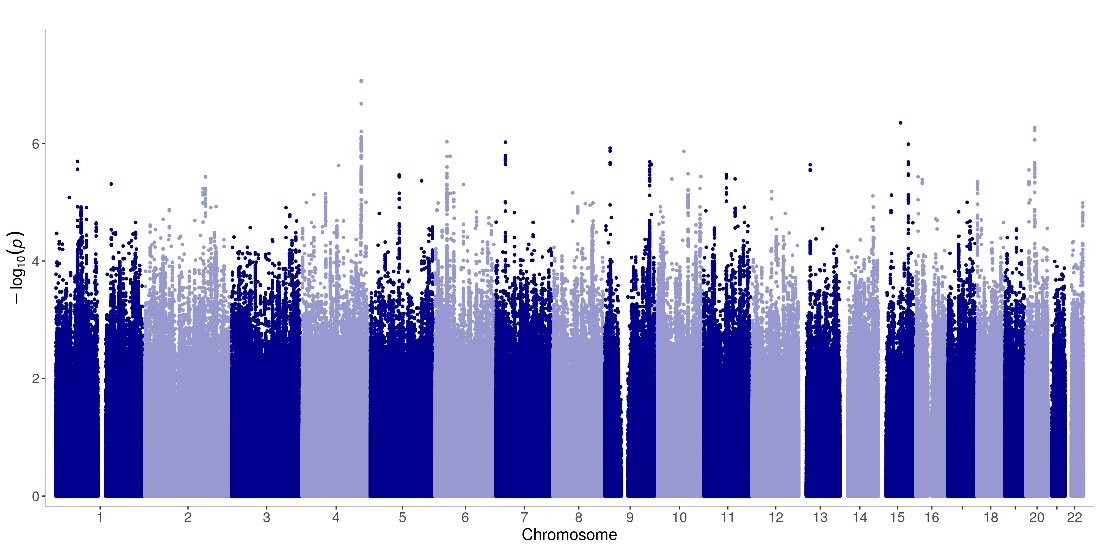


Supplementary Figure 14: Scree plot to determine factor number (n=5)


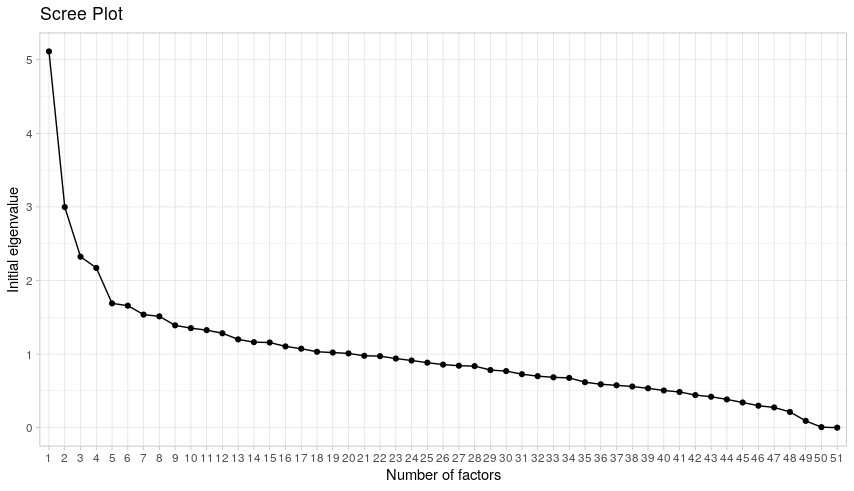


Supplementary Figure 15: Correlogram visualizing the correlation between MLTC latent factors and the number of self-reported diseases.


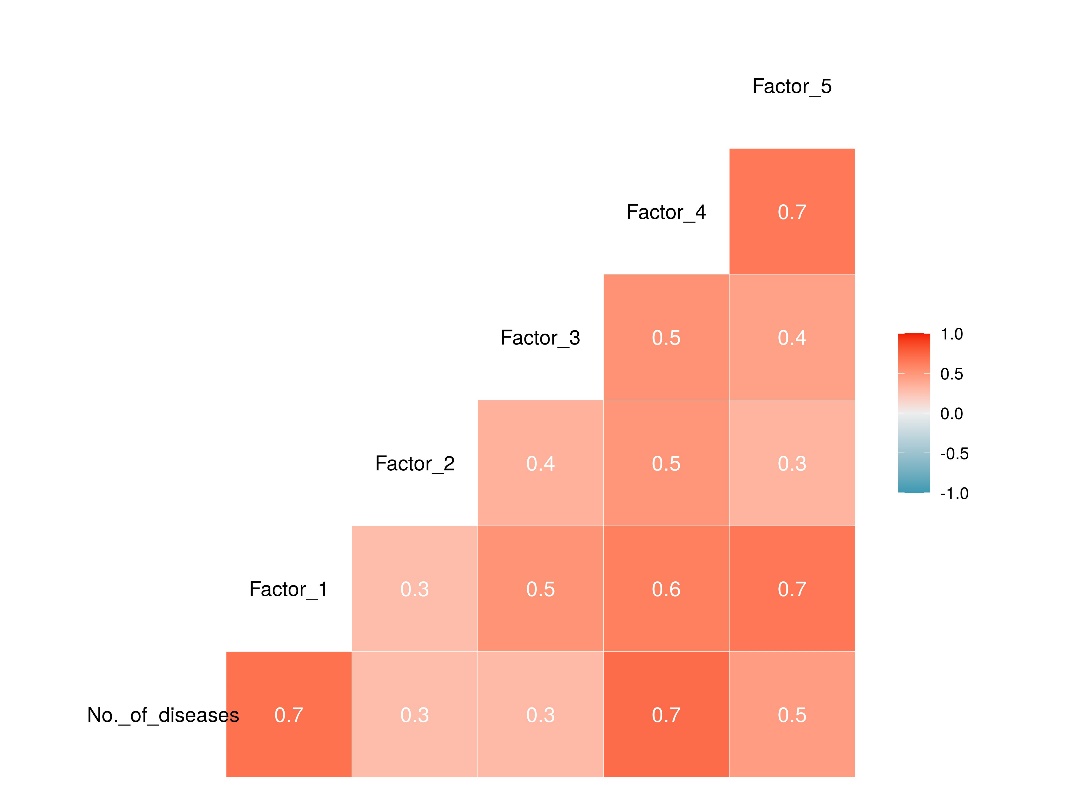


Supplementary Figure 16: Association between latent MLTC factors derived from factor analysis and PRS of different diseases.


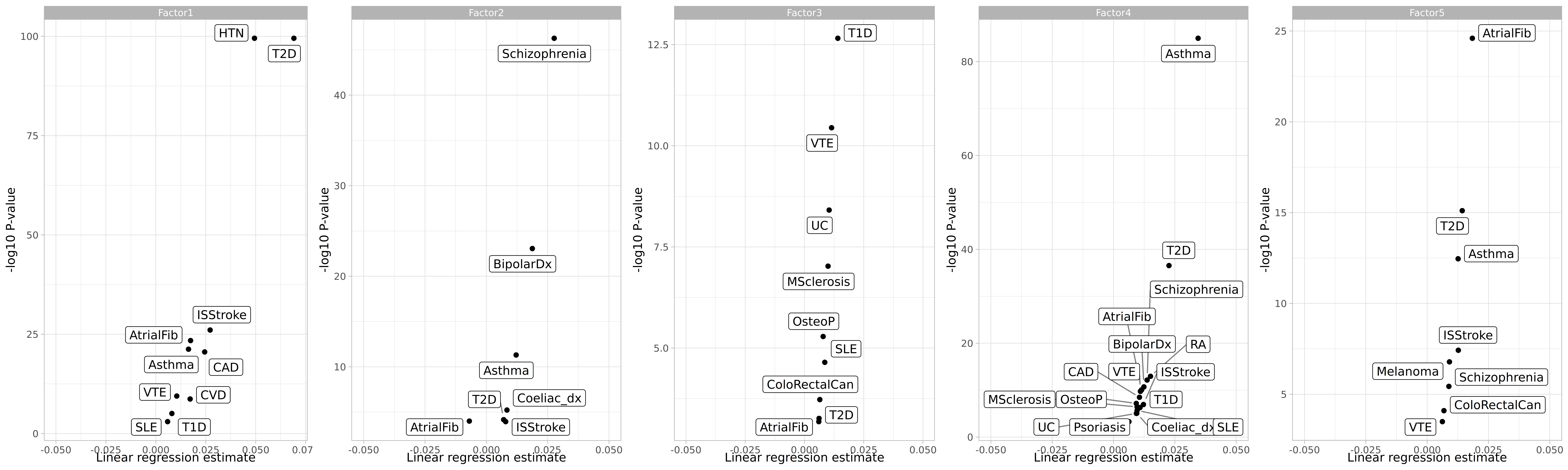


(HTN: hypertension PRS, T2D: Type 2 diabetes PRS, CAD: coronary artery disease PRS, CVD: cardiovascular disease PRS, SLE: Systemic Lupus Erythematosus PRS, VTE: venous thromboembolic disease PRS, T1D: type 1 diabetes PRS, UC: ulcerative colitis PRS, MSclerosis: multiple sclerosis PRS, RA: rheumatoid arthritis)

Supplementary Figure 17: Factor 1 GWAS Manhattan plot


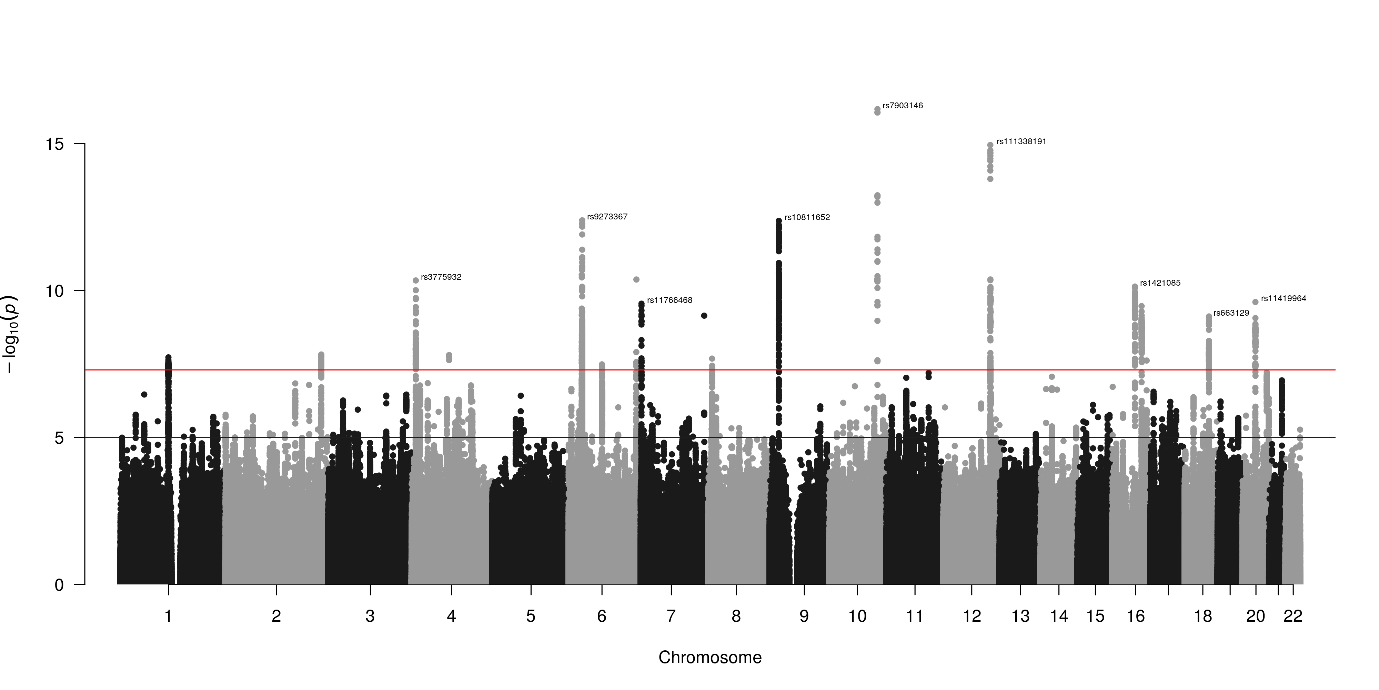


Supplementary Figure 18: Factor 2 GWAS Manhattan plot


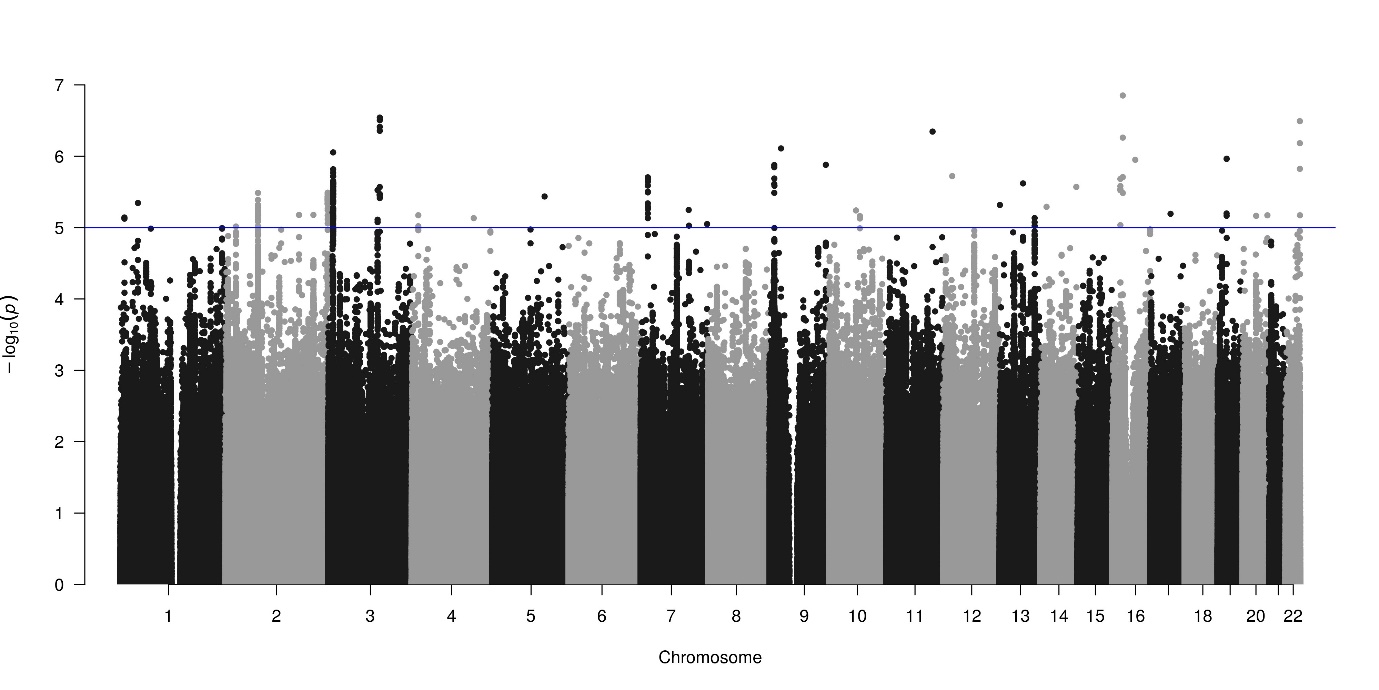


Supplementary Figure 19: Factor 3 GWAS Manhattan plot


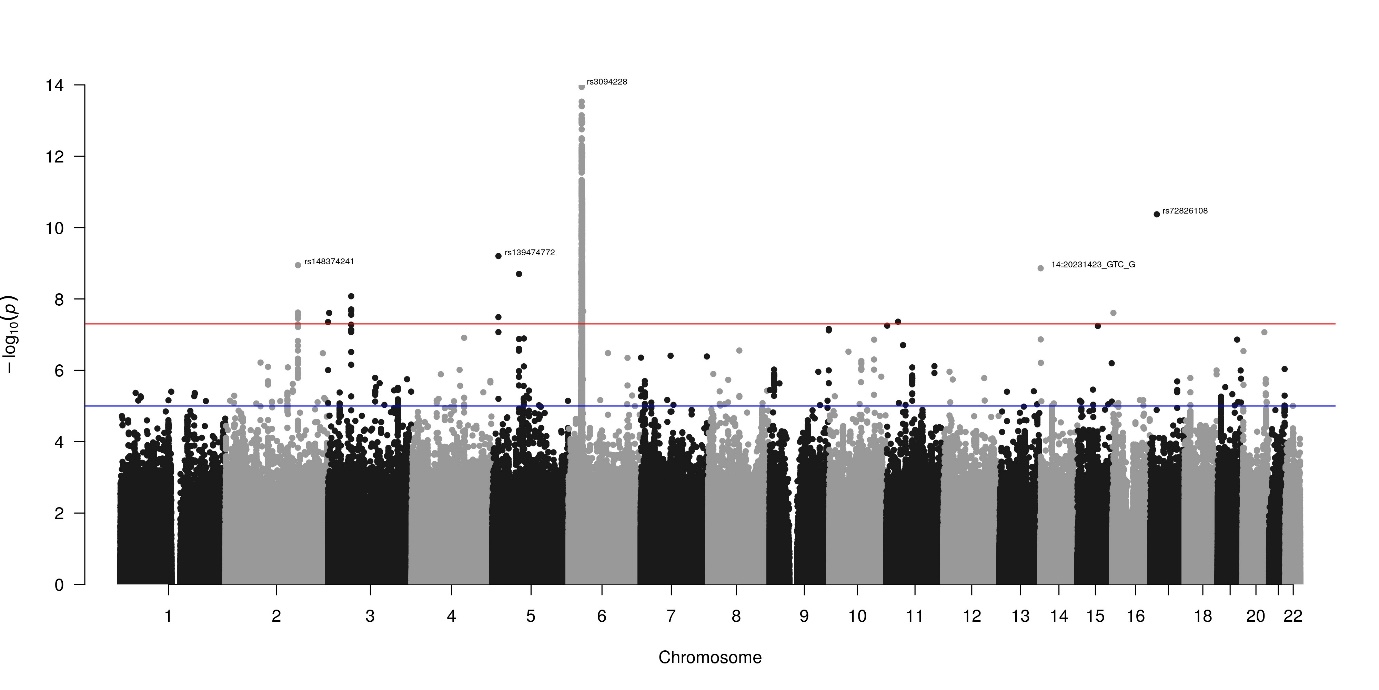


Supplementary Figure 20: Factor 4 GWAS Manhattan plot


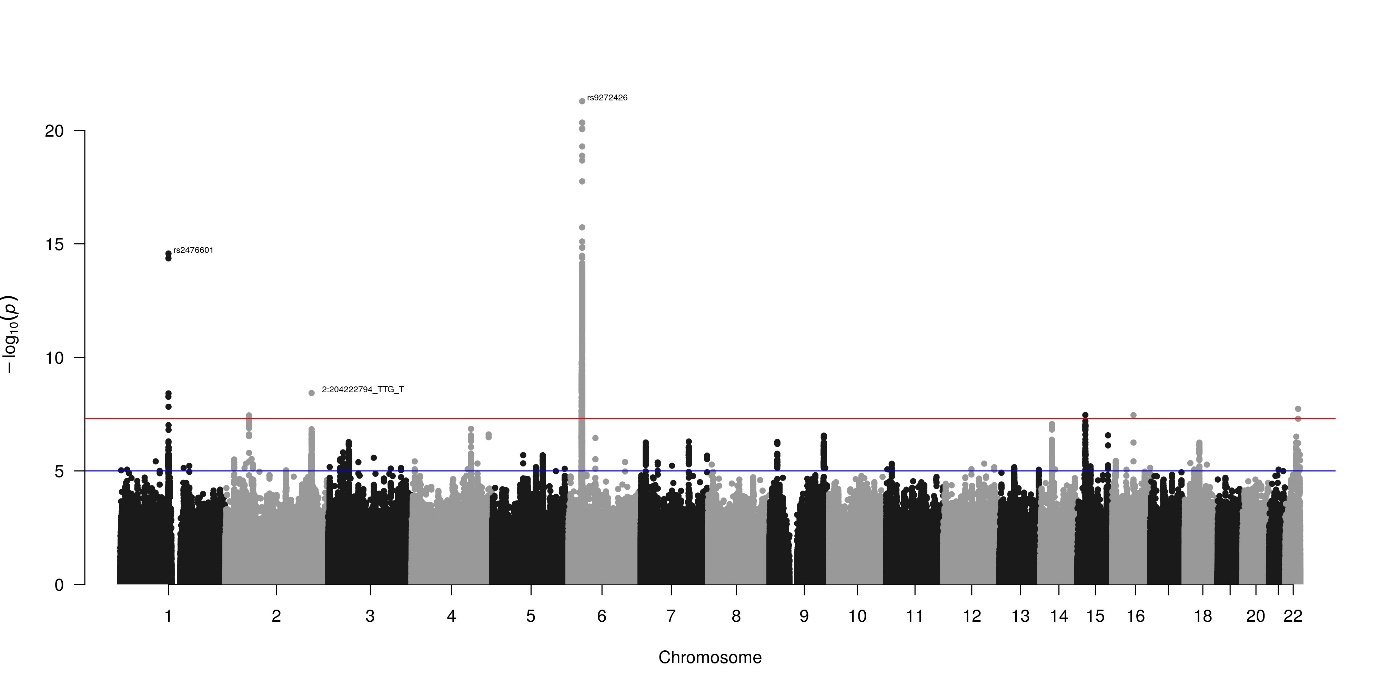


Supplementary Figure 21: Factor 5 GWAS Manhattan plot


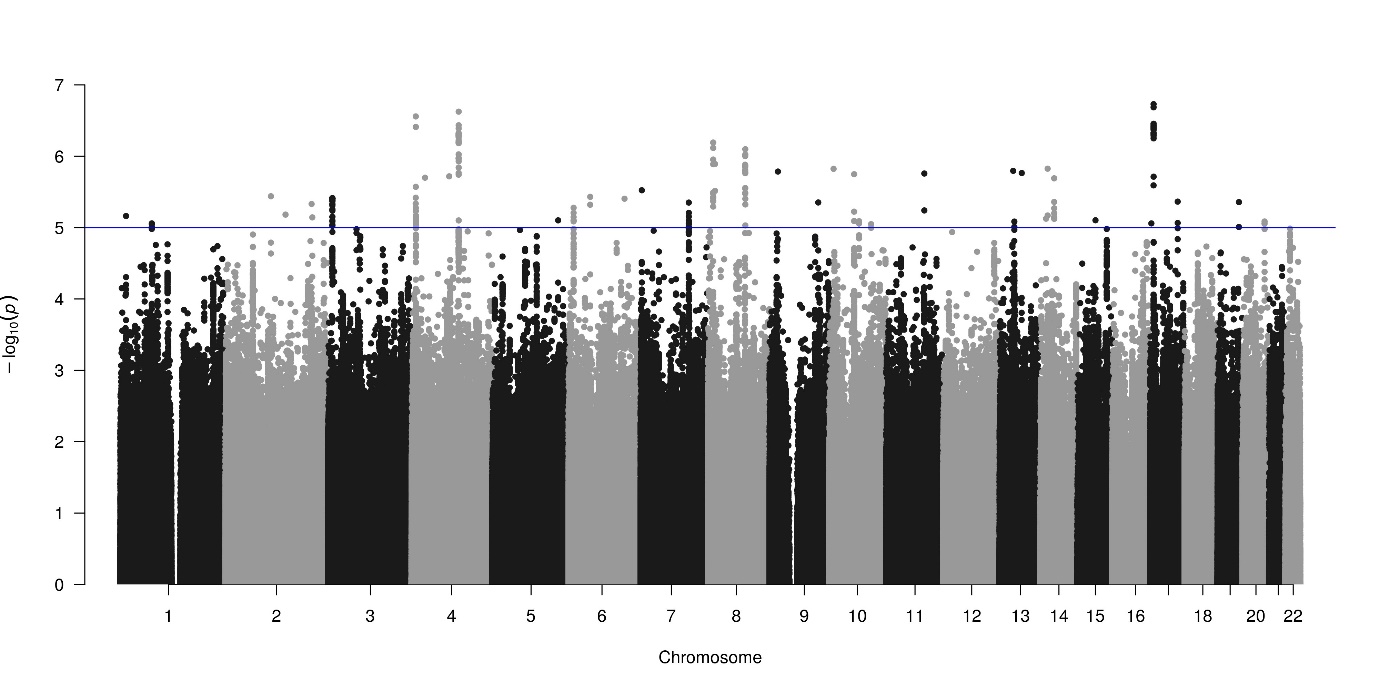


Supplementary Table 1: Characteristics of the study population complex MLTC group and non-complex MLTC group

| **Variable** | **N** | **Non-complex MLTC**  **N = 299,404*^1^*** | **Complex MLTC**  **N = 37,650*^1^*** | **p-value***^2^* |
| --- | --- | --- | --- | --- |
| **Age (years)** | 337,054 | 58 (50, 63) | 61 (56, 65) | <0.001 |
| **Sex** | 337,054 |  |  | <0.001 |
| Female |  | 159,950 (53%) | 21,032 (56%) |  |
| Male |  | 139,454 (47%) | 16,618 (44%) |  |
| **HbA1c(%)** | 321,212 | 35.0 (32.6, 37.4) | 37.0 (34.0, 41.1) | <0.001 |
| **BMI (Kg/m2)** | 335,963 | 26.5 (24.0, 29.5) | 28.8 (25.7, 32.8) | <0.001 |
| **LDL-c (mmol/L)** | 320,738 | 3.56 (3.00, 4.15) | 3.26 (2.64, 3.95) | <0.001 |
| **HDL-c(mmol/L)** | 294,162 | 1.41 (1.19, 1.69) | 1.31 (1.10, 1.58) | <0.001 |
| **Triglyceride (mmol/L)** | 321,080 | 1.47 (1.04, 2.12) | 1.70 (1.20, 2.42) | <0.001 |
| **CRP (mmol/L)** | 320,647 | 1.26 (0.63, 2.59) | 1.99 (0.96, 4.18) | <0.001 |
| **Creatinine (mmol/L)** | 321,179 | 71 (62, 81) | 71 (61, 83) | <0.001 |
| **Number of diseases** | 337,054 | 1.00 (0.00, 1.00) | 3.00 (3.00, 4.00) | <0.001 |
| **No: of Body system** | 337,054 | 1.00 (0.00, 1.00) | 3.00 (3.00, 4.00) | <0.001 |
| *^1^* Median (IQR); n (%) | | | | |
| *^2^* Wilcoxon rank sum test; Pearson’s Chi-squared test | | | | |

**Supplementary text 1: FUMA setting details:**

Post-GWAS functional annotation and gene mapping were conducted using FUMA v1.5.2, incorporating MAGMA v1.08, GWAS catalog version e0_r2022-11-29, and ANNOVAR (2017-07-17). The GWAS summary statistics were derived using the GRCh37/hg19 genome build. The total sample size for the analysis was N = 337,054, with LD calculated using the UK Biobank (UKB/release2b) reference panel, specifically the White British subset (WBrits_10k). Genome-wide significant SNPs were defined using a p-value threshold of 5 × 10⁻⁸, and SNPs in LD with lead SNPs were included using an r² threshold of 0.6, with further pruning at r² < 0.1 to define independent lead SNPs. LD blocks were merged if located within 250 kb. The minimum MAF threshold was set to ≥ 0.01.

MAGMA gene analysis was performed using a gene window of +2 kb upstream and −1 kb downstream of genes, and tissue-specific gene expression data from GTEx v8, including both general average expression and tissue-specific average expression (log2 TPM), was used for tissue enrichment analysis. Positional mapping of SNPs to genes was enabled, using a ±10 kb window around gene boundaries. A functional CADD score threshold of >12.37 was applied to prioritize potentially deleterious variants, with no specific Roadmap (RDB) or chromatin state filtering used.
eQTL mapping was also enabled, using data from GTEx v8, specifically from EBV-transformed lymphocytes and Whole Blood. Significant eQTLs (FDR ≤ 0.05) were mapped to genes within 1 Mb of the transcription start site (TSS), and a CADD score filter of 12.37 was applied. Chromatin interaction mapping was not performed in this analysis.

^##^ADMISSION Research Collaborative Consortium members

Victoria Bartle^5^**,**Rachel Cooper^2,3^**,**Ray Holding^5^,Tom Marshall^6^,Fiona E Matthews^7^,Paolo Missier^7^,Chris Plummer^3,8^,Sian M Robinson^2,3^,Elizabeth Sapey^9,10^,Thomas Scharf^4^,Mervyn Singer^11,12^,James MS Wason^13^

5. Public Co-Investigator, ADMISSION Research Collaborative, Newcastle upon Tyne, UK

6. Institute of Applied Health Research, University of Birmingham, Birmingham, UK

7. Research and Enterprise Office, University of Hull, Hull, UK

8.Digital Services, Newcastle upon Tyne Hospitals NHS Foundation Trust, Newcastle upon Tyne, UK

9.PIONEER Hub, University of Birmingham, Birmingham, UK.

10.Institute of Inflammation and Ageing, University of Birmingham, Birmingham, UK.

11.University College London Hospitals NHS Foundation Trust, London, UK

12.Bloomsbury Institute for Intensive Care Medicine, University College London, London, UK

13.Biostatistics Research Group, Population Health Sciences Institute, Newcastle University, Newcastle upon Tyne, UK
