# Supplementary material for: Exploring the genetic architecture of multiple long-term conditions using a genome-wide association study in the UK Biobank population
Source: Sci Rep. 2025 Dec 6;15:44096. doi: 10.1038/s41598-025-27839-4 (PMC12715236; doi:10.1038/s41598-025-27839-4)

Supplementary File provides the GWAS results based on MLTC GWAS summary statistics adjusted for λ, and adjusted p-values.

Figure A: Gene-based test Manhattan plot from GWAS of MLTC **(with adjusted p values based on λ)** defined as having 2 or more diseases from 51 disease list. (Input SNPs were mapped to 19077 protein-coding genes. Genome-wide significance (red dashed line in the plot) was defined at P = 0.05/19077 = 2.621e-6).
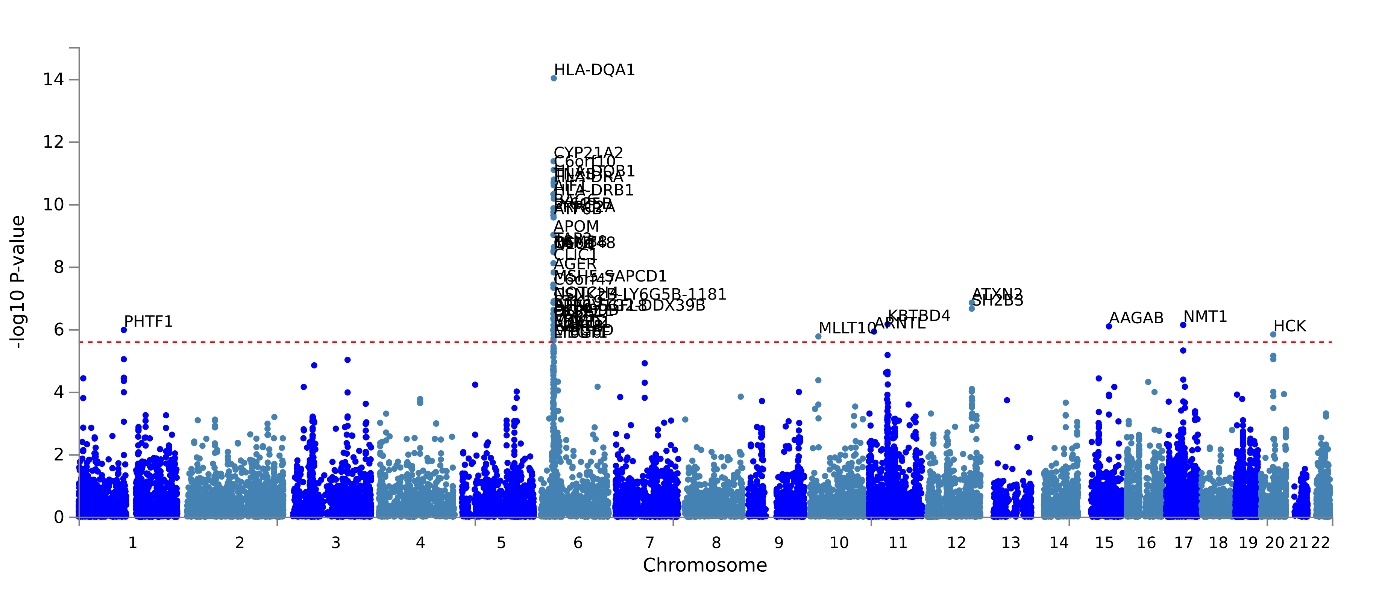


Figure B: Functional consequences of SNPs on genes (FUMA) MLTC GWAS (with adjusted p values based on λ)


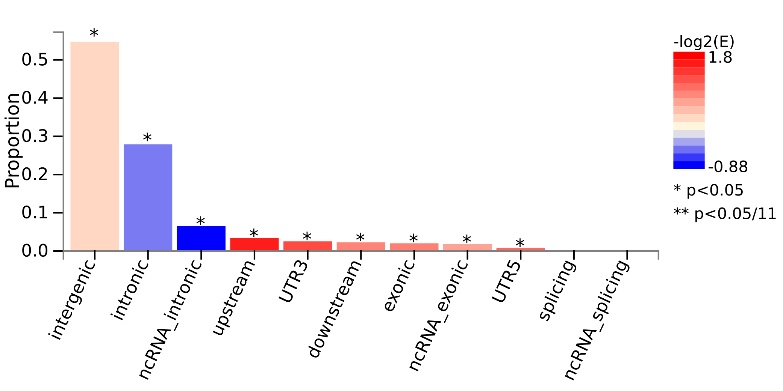


Figure C: Distribution of MLTC GWAS significant SNPs **(with adjusted p values based on λ)**


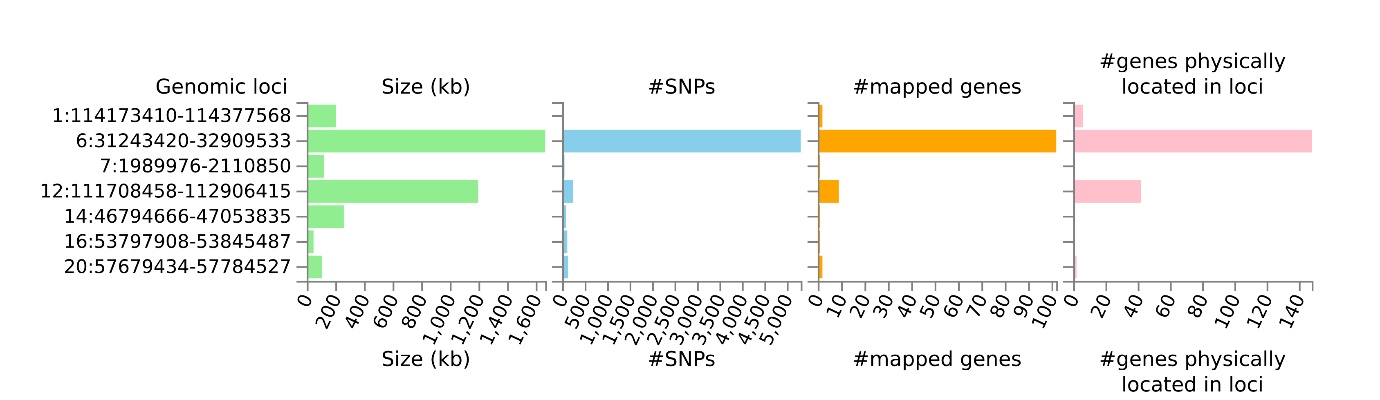


Figure D: Gene set enrichment analysis from FUMA based on MLTC GWAS **(with adjusted p values based on λ)**


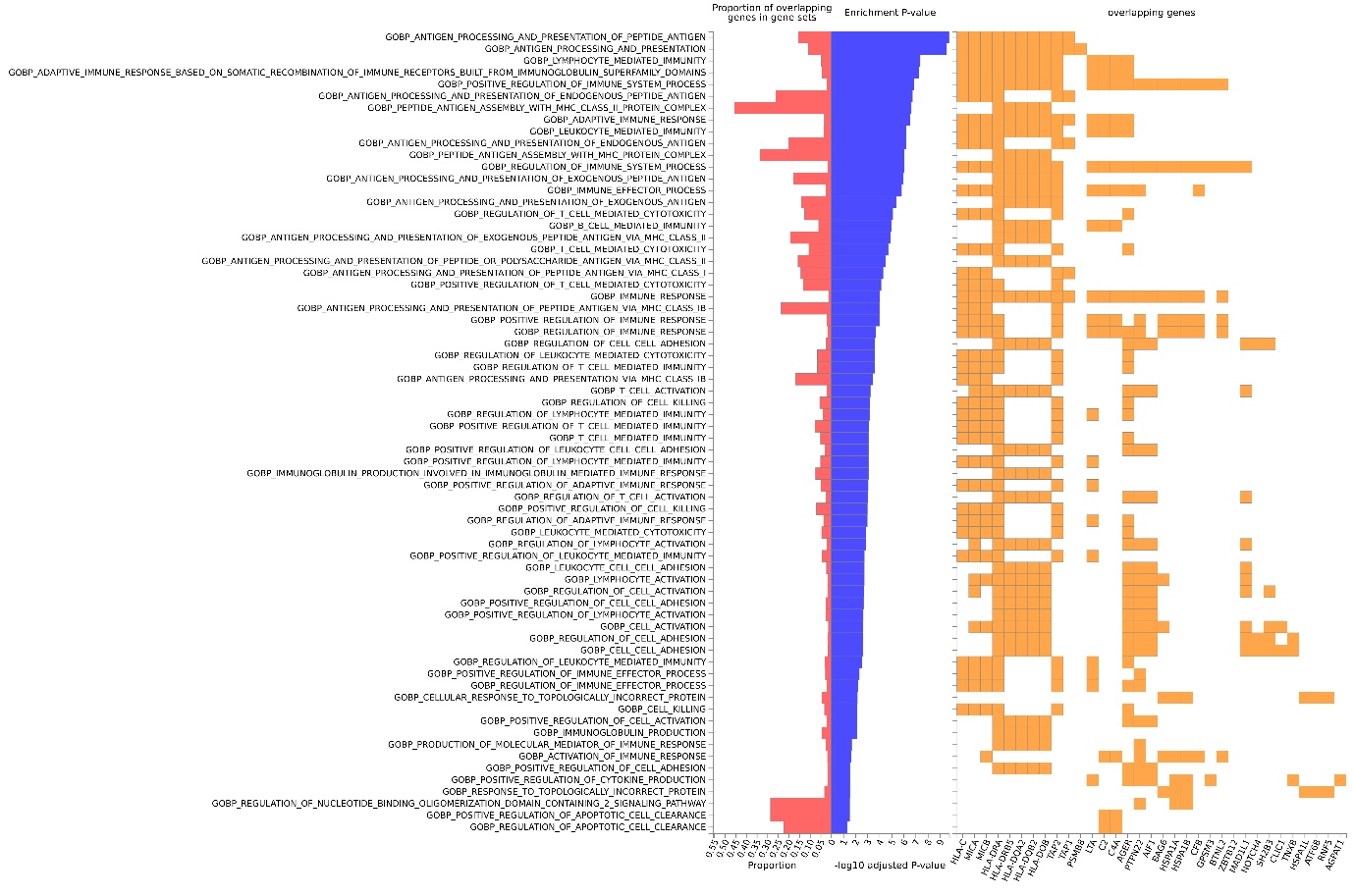

Supplement: Supplementary file 5 — Supplementary Information 5. [file 41598_2025_27839_MOESM5_ESM.docx]
